# A novel splice site mutation of myosin VI in mice leads to stereociliary fusion caused by disruption of actin networks in the apical region of inner ear hair cells

**DOI:** 10.1371/journal.pone.0183477

**Published:** 2017-08-23

**Authors:** Yuta Seki, Yuki Miyasaka, Sari Suzuki, Kenta Wada, Shumpei P. Yasuda, Kunie Matsuoka, Yasuhiro Ohshiba, Kentaro Endo, Rie Ishii, Hiroshi Shitara, Shin-ichiro Kitajiri, Naomi Nakagata, Hirohide Takebayashi, Yoshiaki Kikkawa

**Affiliations:** 1 Mammalian Genetics Project, Department of Genome Medicine, Tokyo Metropolitan Institute of Medical Science, Tokyo, Japan; 2 Division of Experimental Animals, Center for Promotion of Medical Research and Education, Graduate School of Medicine, Nagoya University, Nagoya, Aichi, Japan; 3 Laboratory of Animal Biotechnology, Department of Bioproduction, Faculty of Bioindustry, Tokyo University of Agriculture, Abashiri, Hokkaido, Japan; 4 Histology Laboratory, Advanced Technical Support Department, Center for Basic Technology Research, Tokyo Metropolitan Institute of Medical Science, Tokyo, Japan; 5 Laboratory for Transgenic Technology, Animal Research Division, Center for Basic Technology Research, Tokyo Metropolitan Institute of Medical Science, Tokyo, Japan; 6 Department of Otolaryngology-Head and Neck Surgery, Graduate School of Medicine, Kyoto University, Kyoto, Japan; 7 Division of Reproductive Engineering, Center for Animal Resources and Development (CARD), Kumamoto University, Kumamoto, Japan; 8 Division of Neurobiology and Anatomy, Graduate School of Medical and Dental Sciences, Niigata University, Niigata, Japan; Harvard University, UNITED STATES

## Abstract

An unconventional myosin encoded by the myosin VI gene (*MYO6*) contributes to hearing loss in humans. Homozygous mutations of *MYO6* result in nonsyndromic profound congenital hearing loss, DFNB37. Kumamoto shaker/waltzer (*ksv*) mice harbor spontaneous mutations, and homozygous mutants exhibit congenital defects in balance and hearing caused by fusion of the stereocilia. We identified a *Myo6*^c.1381G>A^ mutation that was found to be a p.E461K mutation leading to alternative splicing errors in *Myo6* mRNA in *ksv* mutants. An analysis of the mRNA and protein expression in animals harboring this mutation suggested that most of the abnormal alternatively spliced isoforms of MYO6 are degraded in *ksv* mice. In the hair cells of *ksv*/*ksv* homozygotes, the MYO6 protein levels were significantly decreased in the cytoplasm, including in the cuticular plates. MYO6 and stereociliary taper-specific proteins were mislocalized along the entire length of the stereocilia of *ksv*/*ksv* mice, thus suggesting that MYO6 attached to taper-specific proteins at the stereociliary base. Histological analysis of the cochlear hair cells showed that the stereociliary fusion in the *ksv*/*ksv* mutants, developed through fusion between stereociliary bundles, raised cuticular plate membranes in the cochlear hair cells and resulted in incorporation of the bundles into the sheaths of the cuticular plates. Interestingly, the expression of the stereociliary rootlet-specific TRIO and F-actin binding protein (TRIOBP) was altered in *ksv*/*ksv* mice. The abnormal expression of TRIOBP suggested that the rootlets in the hair cells of *ksv*/*ksv* mice had excessive growth. Hence, these data indicated that decreased MYO6 levels in *ksv*/*ksv* mutants disrupt actin networks in the apical region of hair cells, thereby maintaining the normal structure of the cuticular plates and rootlets, and additionally provided a cellular basis for stereociliary fusion in *Myo6* mutants.

## Introduction

In vertebrates, the inner ear hair cells are crucial to hearing and vestibular function. The mechanically sensitive region of the hair cells is the hair bundle, a group of tightly associated parallel filamentous actin (F-actin)-filled stereocilia, which is required for proper mechanotransduction in response to external forces and for transmitting information about sound and movement [1–3]. The stereocilia insert as a rootlet into a dense filamentous actin mesh structure known as the cuticular plate; the apical end of the hair cells has an F-actin core cytoskeletal architecture [1, 3–5]. The insertion of stereocilia into the cuticular plates shows a tapering morphology, which is important for stereocilia function, because the stereocilia pivot around the taper and move as a unit during mechanical stimulation [2–4].

Several molecules that are functionally associated with the basal region of the stereocilia have been identified in genetic studies of deaf human patients as well as mouse models [6–15]. Among these molecules, one unconventional myosin, myosin VI (MYO6), has been shown to play crucial roles in the constriction and integrity of the stereociliary base. MYO6 is unique among the known myosins because it moves in a direction opposite from that of the other myosins, toward the pointed (minus) end of the F-actin [16, 17]. In vertebrate hair cells, the expression of MYO6 is concentrated in the basal region of the stereocilia, cuticular plate and cytoplasm [5, 6, 18–21]; thus, MYO6 has been predicted to move toward the minus ends of the actin filaments located near the rootlets of the stereocilia [3, 21, 22]. The Snell’s waltzer (*Myo6*^*sv*^) mouse was the first reported animal model of a *Myo6* mutant that exhibits shaker/waltzer behavior and congenital profound hearing loss, which is caused by the fusion and taper loss of the stereocilia [6, 23]. *Myo6*^*sv*^ is a null mutation that leads to the complete loss of MYO6 [6]. The stereociliary fusion in *Myo6*^*sv*^ mice is caused by the absence of MYO6 in the hair cells, probably at the bases of the stereocilia and/or on the apical surfaces of the cuticular plates. Notably, mutations in human *MYO6* have been shown to cause recessive DFNB37 [24] and dominant DFNA22 [25] nonsyndromic hearing loss.

MYO6 appears to act as a cargo transporter that moves critical regulatory components to the bases of the stereocilia and the apical surfaces of the cuticular plate. The chloride intracellular channel 5 (CLIC5), protein tyrosine phosphatase receptor Q (PTPRQ), radixin (RDX) and taperin (TPRN) proteins are localized at the tapered region of the stereocilia and/or apical surface of the cuticular plate in mice [8–10, 21, 22]. In the fused stereocilia in *Myo6*^*sv*^ mice, CLIC5, PTPRQ and RDX do not localize to the tapered region and/or the apical surface but instead are broadly localized along the entire length [21, 22]. Mutant mice for *Clic5*, *Ptprq* and *Rdx* have been established and exhibit balance and hearing defects caused by the fusion and taper loss of the stereocilia, a phenotype similar to that of the *Myo6*^*sv*^ mice [8, 9, 21, 22, 26], thus suggesting that the regulation of the localization of these proteins to the tapered region in the stereocilia and/or the apical surface of the cuticular plate by MYO6 is required for the constriction and integrity of the stereociliary base.

As mentioned above, MYO6 is essential for normal stereocilia architecture. However, the detailed processes and mechanisms of stereociliary fusion and taper loss in *Myo6* mutant mice remain unknown. In this study, we report a novel spontaneous *Myo6* mutation, Kumamoto shaker/waltzer (*ksv*), which leads to balance and hearing defects. Although we determined that the *ksv* mutation is a loss-of-function mutation, the processes and phenotypic features of stereociliary fusion in *ksv* mice were highly similar to those of MYO6-null *Myo6*^*sv*^ mice. Here, we demonstrate that an appreciable decrease in MYO6 on the apical surfaces of hair cells and the mislocalization of MYO6 along the length of the stereocilia leads to stereociliary fusion, as a result of the disruption of the actin networks in the stereociliary base, including the rootlet and cuticular plate.

## Materials and methods

### Ethics statement

All procedures involving animals met the guidelines described in the Proper Conduct of Animal Experiments as defined by the Science Council of Japan and were approved by the Animal Care and Use Committee on the Ethics of the Tokyo Metropolitan Institute of Medical Science (Igakuken) (Approval numbers: 13036, 14081, 15046 and 16064).

### Mice

The *ksv* mutants exhibiting shaker/waltzer behavior were first identified in a litter of B6;129S4-*Gt(ROSA)26Sor*^*tm2Dym*^/J strain mice at the Center for Animal Resources and Development (CARD) of Kumamoto University. This strain possesses a disrupted human placental alkaline phosphatase (*ALPP*) transgene inserted into the Gt(ROSA)26Sor locus. F_1_ *ksv* mutants of the C57BL/6J (B6J) (CLEA Japan, Tokyo, Japan) strain were backcrossed to B6J mice for 10 generations at the Igakuken. The +/*ksv* heterozygous and *ksv/ksv* homozygous offspring of each breeder pair, consisting of a +/*ksv* female and a *ksv/ksv* male, were used for all experiments. *Myo6* null mutant (B6J-*Myo6*^-/-^) mice, which have been previously reported as *Myo6*^*rsv*^ [20], were maintained at the Igakuken. To produce genetic crosses, JF1/Ms (JF1) and MSM/Ms strains were obtained from the National Institute of Genetics and maintained at the Igakuken. All mice were maintained under specific-pathogen-free (SPF) conditions for temperature (23 ± 1°C), relative humidity (50 ± 10%) and light/dark cycles (12 hour/12 hour) and were given food and water ad libitum. The methods for euthanasia included cervical dislocation and carbon dioxide (CO_2_) inhalation.

### Behavioral and hearing tests

The shaker/waltzer behavior of the B6J (+/+) and *ksv*/*ksv* mice was measured using an open-field behavioral test. Mice were placed in a 50 cm × 40 cm × 50 cm (W × H × L) open field to quantify their behaviors, as previously described [27]. The hearing ability of the mice was measured via the auditory brain stem response (ABR). An ABR workstation (TDT System III, TDT, Alachua, FL, USA) was used to test mice for ABR thresholds, as previously described [27]. The ABR was detected using tone-pip stimuli for each maximum sound pressure level at 4, 8, 16 and 32 kHz.

### Electron microscopy

Scanning electron microscopy (SEM) of the cochlear stereocilia was performed as previously described [27] in at least four ears from +/+ (postnatal day 30 (P30)), +/*ksv* (P0 and P4) and *ksv/ksv* (P0, P2, P4, P7, P14 and P30) mice to analyze cochlear hair cells, and from +/+ (P7 and P14) and *ksv/ksv* (P7 and P14) to analyze vestibular hair cells, by using a Hitachi S-4800 field emission SEM instrument (Hitachi High-Technologies Corporation, Tokyo, Japan) at an accelerating voltage of 10 kV.

For transmission electron microscopy (TEM), +/+ and *ksv*/*ksv* mice at P1 were perfused through the heart with a buffer containing 2% paraformaldehyde (PFA)/2.5% glutaraldehyde (GA) in 0.1 M phosphate buffer (PB) (pH 7.4). Immediately after the perfusion, the inner ears were removed from the heads of the mice, and small holes were made at the tops of the cochleae using a 27 gauge needle. The holes of the inner ears were gently flushed with a 2% PFA/2.5% GA fixative solution and then postfixed overnight at 4°C. The inner ears were then rinsed two times in 0.1 M phosphate-buffered saline (PBS) for 10 min and decalcified in 10% EDTA dipotassium salt for three weeks at 4°C. After decalcification, the samples were washed two times in 0.1 M PBS for 10 min and refixed with 2.5% GA in 0.1 M cacodylate buffer (CB) for 30 min at 4°C. Following the fixation, the samples were washed three times in 4.5% sucrose/0.05% CaCl_2_/0.025% NaN_3_/0.1 M CB for 5 min and postfixed with 2% osmium tetroxide in 0.1 M CB for 2 hr. Then, the samples were dehydrated in an ascending ethanol series (50% for 5 min at 4°C, 70% for 15 min at 4°C, 80% for 15 min at 4°C, 90% for 15 min at room temperature (RT), and 100% for 20 min at RT (three times), equilibrated three times in propylene oxide (PO) for 10 min, embedded in PO/EPON 812 (TAAB Laboratories Equipment, Aldermaston, UK) for 2 hr, and cured in EPON 812 resin for 48 hr at 65°C. The embedded samples were re-sectioned into 4-μm serial semi-thin sections, and the target regions were identified under a light microscope and then re-embedded. Ultrathin sections were stained with uranyl acetate for 30 min and lead citrate for 5 min and examined under a JEM-1400 transmission electron microscope (JEOL, Ltd., Tokyo, Japan).

### Genetic mapping, allelism testing and mutation analyses

Genetic mapping of the *ksv* mutation was performed by using intersubspecific backcross progeny from (JF1 × B6J-*ksv/ksv*) F_1_ × B6J-*ksv/ksv* (*n* = 15) and (MSM × B6J-*ksv/ksv*) F_1_ × B6J-*ksv/ksv* (*n* = 39). Progeny were phenotyped on the basis of visible behavior, according to the easily identifiable characteristics of normal (+/*ksv*) or shaker/waltzer (*ksv*/*ksv*) behavior. DNA samples from 54 backcrossed mice were genotyped by using 14 MIT markers on chromosome 9 and the *D9Nok14* locus [20], which is located in intron 31 of *Myo6* (Ensembl GRCm38.p4, Chr 9: 80,300,769–80,300,891). The PCR conditions used for genotyping were as previously described [27]. For the allelism test of the mutant allele of *Myo6*, +/*ksv* heterozygous mice were crossed to *Myo6*^-/-^ homozygous mice [20]. The F_1_ offspring were phenotyped on the basis of visible behavior, according to the identifiable characteristics of normal (+/-) or shaker/waltzer (*ksv*/-) behavior. The *ksv* mutation in *Myo6* was detected by Sanger sequencing. Mutation analysis was performed on genomic DNA and cDNA in +/+, +/*ksv* and *ksv/ksv* mice by using previously described primers and methods [20].

### Reverse transcriptase polymerase chain reaction

The total RNA was isolated from the cochlea and vestibule of +/+, +/*ksv* and *ksv/ksv* mice at P30 using a PureLink RNA Mini Kit (Thermo Fisher Scientific, Waltham, MA, USA) according to the manufacturer’s protocol. We carried out a semiquantitative reverse transcriptase polymerase chain reaction (RT-PCR) using KOD FX Neo (TOYOBO, Osaka, Japan) and the *Myo6* (Myo6_c1077F and Myo6_c1687R) and *Gapdh* (Gapdh_c326F and Gapdh_c1128R) primer sets (S1 Table). The amplification conditions were as follows: 94°C for 2 min, followed by 35 cycles of 98°C for 10 s and 68°C for 90 s; the products were then subjected to 2% agarose gel electrophoresis. The PCR products were purified by agarose gel electrophoresis using a QIAquick Gel Extraction Kit (Qiagen, Valencia, CA, USA), subcloned into a pT7 Blue T-vector (Merck Millipore, Darmstadt, Germany), and sequenced. Quantitative RT-PCR (qRT-PCR) was performed using a QuantiTect SYBR Green PCR Kit (Qiagen) and the primer sets for *Myo6* (Myo6_c98F and Myo6_c245R) and for *Gapdh* (Gapdh_c251F and Gapdh_c374R) listed in S1 Table according to the manufacturer’s protocol. The products were analyzed on a LightCycler 480 instrument (Roche Diagnostics). The signal values were normalized to the *Gapdh* median signals, and the geometric mean values of the target signals were calculated in triplicate. The expression levels of the genes in +/+ mice were assigned an arbitrary value of 1 for comparison.

### Antibodies

We also generated an anti-MYO6 (N-ter) rabbit polyclonal antibody to a peptide (HPTDGFQMGNIVDIG+C) in the N-terminal region of the MYO6 peptide from aa 11 to 25. The anti-PTPRQ antibody was generated against the synthetic peptide of the amino acid sequence (ENDIFVRTPEDEPES) corresponding to a peptide described by Sakaguchi et al [21]. The anti-TRIO and F-actin binding protein isoform 5 (TRIOBP-5) antibody was previously described [28]. The primary antibodies specific for MYO6 (C-ter), TPRN, β-tubulin, α-tubulin, α-2 spectrin (spectrin alpha, non-erythrocytic 1: SPTAN1) and sorting nexin 9 (SNX9) used in this study were commercially obtained and had been characterized in previous studies [4, 12, 15, 18–20, 22, 29–32]. The primary and secondary antibodies used in this study are listed in S2 Table.

### DNA constructs, cell culture, transfection, and immunocytochemistry

The full-length *Myo6* gene was amplified by PCR from the cochlear cDNA of +/+ and *ksv*/*ksv* mice by using EcoRI_Myo6_c31F and Myo6_c3860R_SalI primers (S1 Table). The amplified fragments were purified and cloned into the *Eco*RI/*Sal*I site of the pAcGFP1-C1 (Clontech, Mountain View, CA, USA), pDsRed-Monomer-C1 (Clontech) and pCAGGS (gift from Dr. H. Niwa) [33] mammalian expression cloning vectors, and MYO6-wild-type and p.G360_F460del constructs were selected. Green fluorescent protein (GFP)-, *Discosoma* sp. red fluorescent protein (DsRed)- and FLAG-tagged MYO6-p.E461K constructs were created with a QuikChange II XL Site-Directed Mutagenesis Kit (Agilent Technologies, Santa Clara, CA, USA) according to the manufacturer's protocol. Simian fibroblast COS7 cells were plated on culture dishes and maintained at 37°C and 5% CO_2_ in Dulbecco’s modified Eagle’s medium (DMEM) with 10% fetal bovine serum (FBS). The cultures were transfected with plasmid DNA with Lipofectamine LTX & Plus reagent (Thermo Fisher Scientific) and Lipofectamine 2000 reagent (Thermo Fisher Scientific) according to the manufacturer's protocol. For immunocytochemistry, the cells were fixed with 4% PFA for 1 hr, then permeabilized with 0.25% Triton X-100 for 10 min and washed three times for 5-min each in 0.1 M PBS. The nonspecific binding sites were then blocked with 0.5% Blocking Reagent (Roche Molecular Biochemicals, Indianapolis, IN, USA) for 1 hr at RT. After blocking, the samples were incubated with anti-MYO6 (N-ter) and/or -FLAG antibodies (S2 Table) diluted in Can Get Signal Immunostain Solution B (TOYOBO) overnight at 4°C. Subsequently, the samples were washed three times for 5 min each in PBS and incubated with Alexa Fluor 488- and 568-conjugated secondary antibody (S2 Table), 633-conjugated phalloidin (8 units/ml, Thermo Fisher Scientific) and DAPI (5 μg/ml, Thermo Fisher Scientific) in Can Get Signal Immunostain Solution B for 1 hr at RT. The samples were then washed three times for 5 min each in PBS, mounted onto slides using PermaFluor (Thermo Fisher Scientific), and imaged using a Zeiss LSM780 confocal microscope (Carl Zeiss, Jena, Germany).

### Immunohistochemistry

The immunohistochemistry of the whole-mount preparations was performed as described in our previous study [27] for staining with anti-MYO6 (N-ter), MYO6 (C-ter), TPRN, SNX9 and SPTAN1 antibodies, as described by Sakaguchi et al. [21] for staining with anti-PTPRQ antibody, and as described by Kitajiri et al. [28] for staining with anti-TRIOBP-5 antibody in at least four ears of +/+, +/*ksv* and *ksv*/*ksv* mice at each tested age. The primary and secondary antibodies used in these experiments are listed in S2 Table. Fluorescence images were obtained using Zeiss LSM780 and LSM510 confocal microscopes (Carl Zeiss) and analyzed with ZEN 2010 software (Carl Zeiss). Except for the quantification of the fluorescence intensity, the samples were z-stack imaged and three-dimensionally deconvoluted using the nearest neighbor and/or regularized inverse filtering algorithms of the ZEN Deconvolution software (Carl Zeiss) to reach confocal-like resolution.

### Western blotting

Proteins were isolated from the inner ears of the +/+, +/*ksv* and *ksv/ksv* mice at P30 using T-PER Tissue Protein Extraction Reagent (Thermo Fisher Scientific) according to the manufacturer's protocol. Fifty micrograms of protein from each tissue was added to an equal volume of 2× sample buffer (100-mM Tris-HCl (pH 6.8), 4% sodium dodecyl sulfate, 20% glycerol, 0.01% bromophenol blue and 5% 2-mercaptoethanol) and heated at 95°C for 5 min. The proteins were separated on a 4–15% Mini-PROTEAN TGX Precast Gel (Bio-Rad, Hercules, CA, USA), transferred to an Immobilon-FL transfer membrane (Merck Millipore), blocked in Odyssey blocking buffer (LI-COR, Lincoln, NE, USA) for 1 hr at RT, and incubated with primary antibodies (S2 Table) diluted in blocking buffer overnight at 4°C. The following primary antibodies were used for Western blotting: anti-MYO6 (C-ter), anti-MYO6 (N-ter) and anti-α-tubulin (S2 Table). After washing with phosphate-buffered saline with Tween 20 (PBST), the membranes were incubated with fluorescently labeled secondary antibodies (S2 Table) for 1 hr at RT. The membranes were scanned, and the fluorescence signals were detected and quantitated using the Odyssey CLx Infrared Imaging System (LI-COR).

### Statistical analysis

All results are presented as the mean ± standard deviation (SD). Differences among multiple groups were analyzed by a one-way ANOVA with the Tukey post hoc multiple comparison test. The two groups were compared using Student’s t-test. GraphPad Prism 5 (GraphPad, San Diego, CA, USA) was used to calculate the column statistics and compute the P values.

## Results

### The *Myo6* mutation causes fusion of stereocilia in *ksv* mice

**Fig 1:**
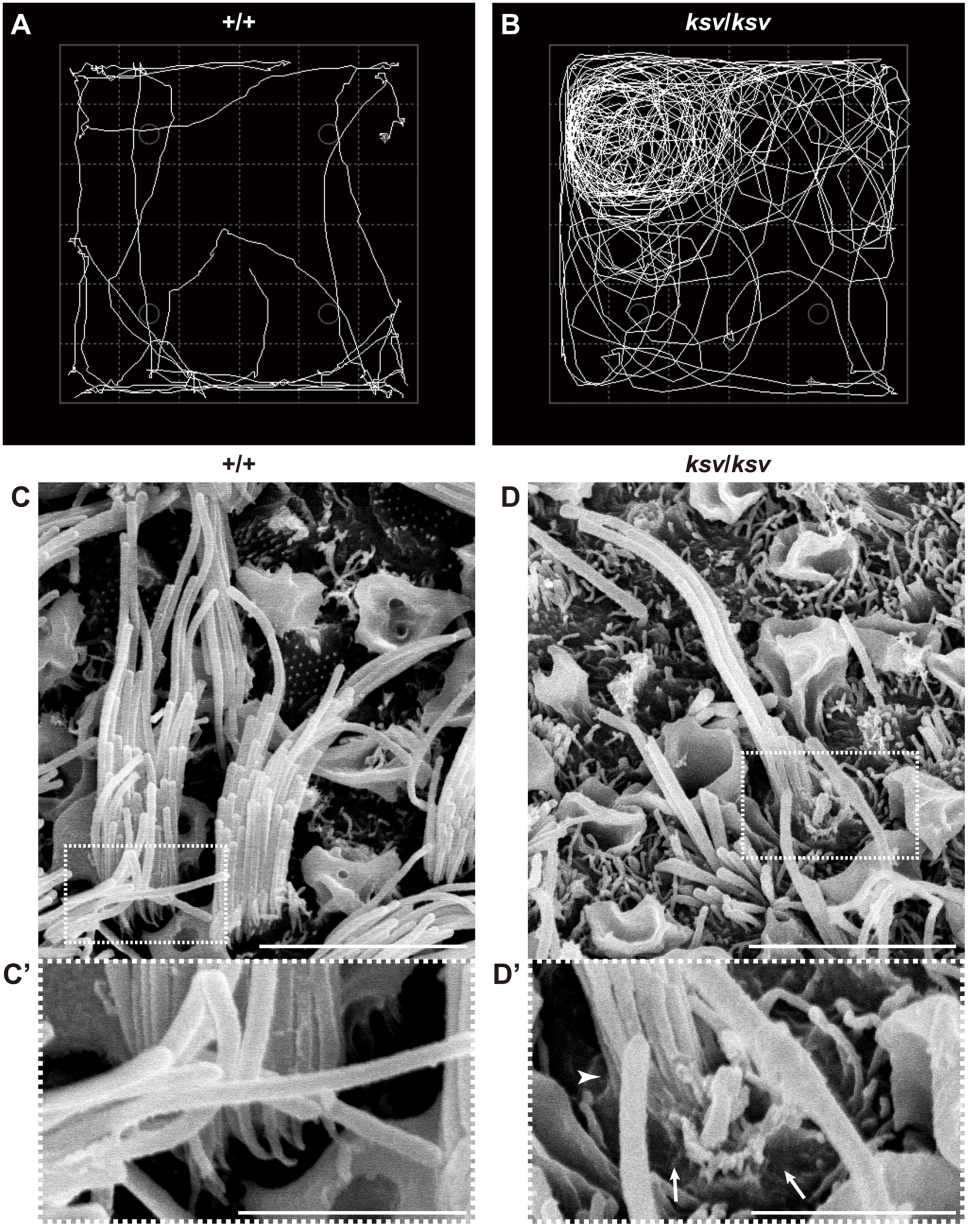
Homozygous *ksv* mutation in mice causes balance defects and stereociliary degeneration of vestibular hair cells. A and B. Representative open-field pathway traces (white curved line) at 1 month of age in wild-type (+/+) (A) and *ksv/ksv* (B) mice. C and D. SEM images of stereocilia from the utricles in the +/+ (C) and *ksv/ksv* (D) mice at P14. Highly magnified images of stereociliary bases (dotted boxes) in C and D are shown in each bottom panel (C’ and D’). Arrows and arrowhead indicate a bulged basal region and a loss of tapered region, respectively, in the stereocilia of *ksv*/*ksv* mice. Scale bars = 5 μm (C and D), and 2 μm (C’ and D’).

**Fig 2:**
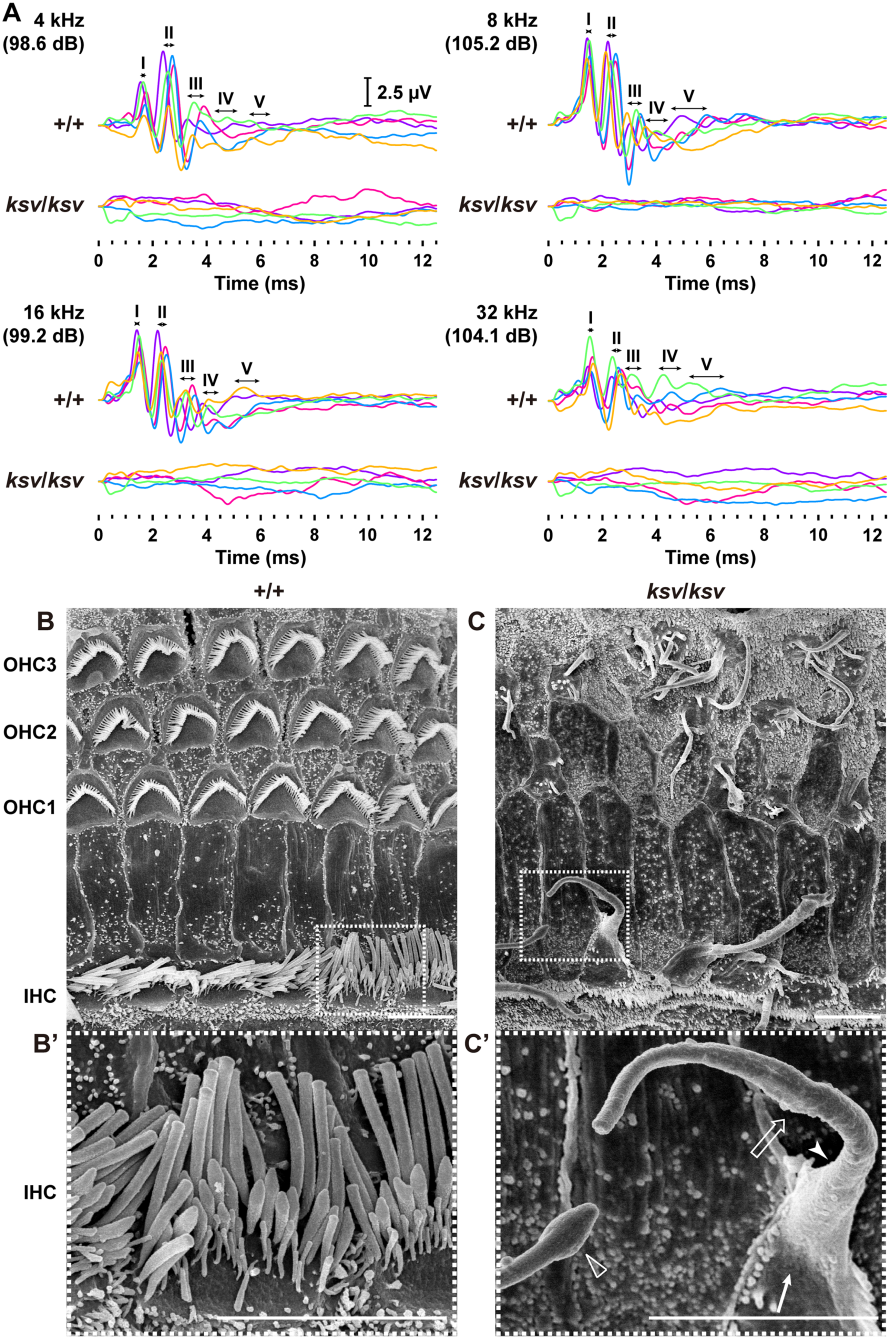
Homozygous *ksv* mutation in mice causes hearing loss and stereociliary fusion of cochlear hair cells. A. ABR waveforms from +/+ and *ksv/ksv* mice at P30. The waveforms from five mice of each genotype represent the ABR to tone-pip stimuli at the highest sound pressure level (dB SPL) at 4, 8, 16 and 32 kHz and are shown in different colors. The locations of ABR peaks I–V are indicated with ranges (two direction arrows) of the negative wave apex in each peak of +/+ mice. B and C. SEM images of stereocilia in the hair cells from the apex area of the cochlea in the +/+ (B) and *ksv/ksv* (C) mice at P30. The highly magnified images in the white dotted boxes of B and C are shown in the bottom panels (B’ and C’). The fused giant stereocilia (open arrow), bulged base (arrow), loss of tapered region (arrowhead) and bulbous tip (open arrowhead) of the stereocilia detected in the IHCs of *ksv*/*ksv* mice (C). Scale bars = 5 μm.

**Fig 3:**
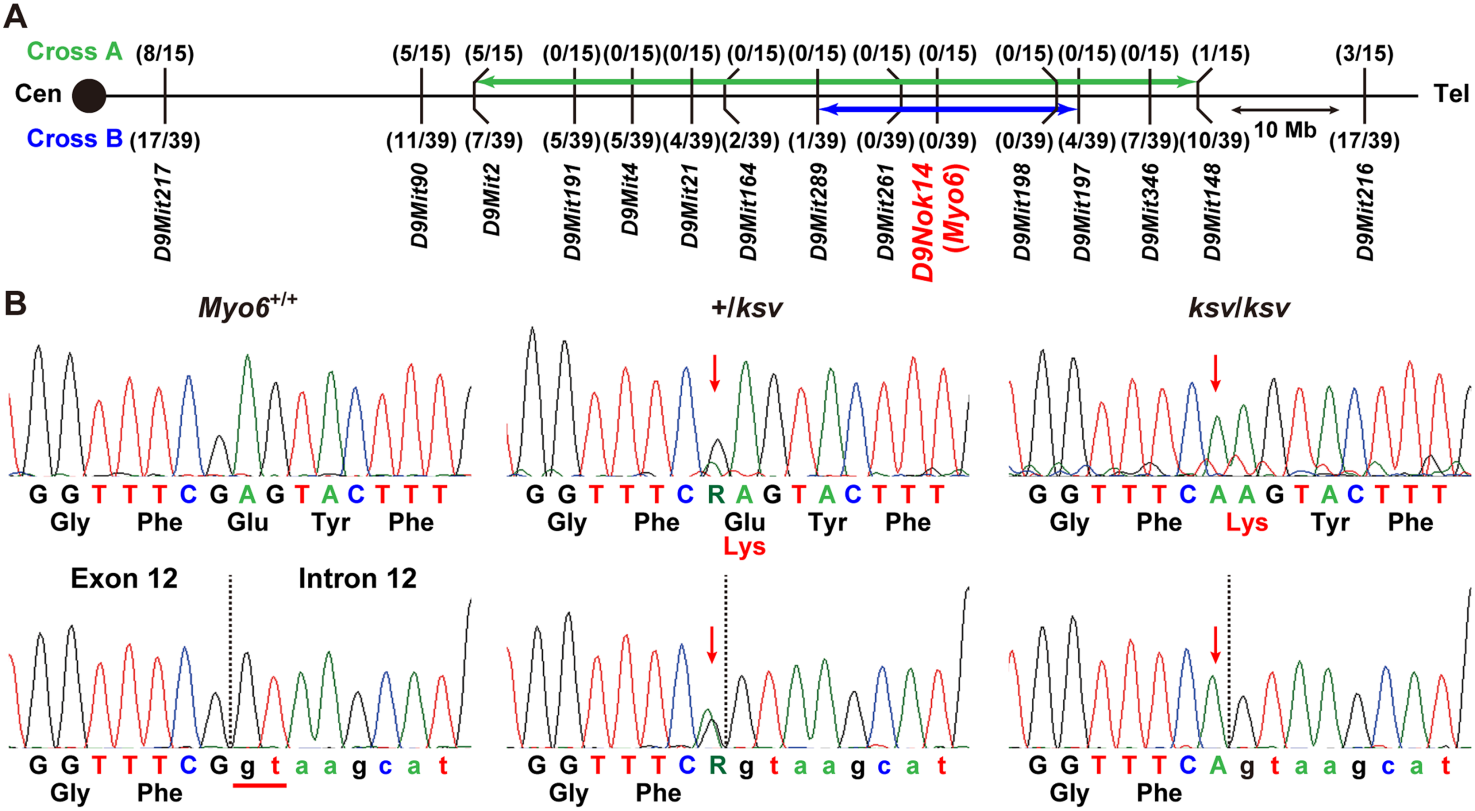
Identification of a missense mutation in *Myo6* of *ksv* mice. A. Genetic map obtained by the phenotyping and genotyping of progeny from two intersubspecific backcrosses, (JF1 × B6J-*ksv*/*ksv*) F_1_ × B6J-*ksv*/*ksv* (Cross A) and (MSM × B6J-*ksv*/*ksv*) F_1_ × B6J-*ksv*/*ksv* (Cross B). Distances between genetic markers given in the Ensembl base pair base locations along chromosome 9 and the number of recombinants per mouse examined are also shown. Green and blue arrows connect recombinant flanking markers used for genotyping of two backcrosses, delineating candidate gene intervals for the *ksv* mutation. *Myo6* was located in the candidate gene interval. B. Identification of a *ksv* mutation in *Myo6*. Sequence analysis of *Myo6* cDNA (top) and genomic DNA (bottom) from *Myo6*^+/+^ (+/+), +/*ksv* heterozygous and *ksv/ksv* homozygous mice. By sequence analysis of the cDNA, a c.1381G>A (p. E461K) missense mutation was detected in *ksv* mice. The G>A mutation occurs one base before the donor site (red line at the left of the bottom panel) of exon 12 in the *Myo6* genomic sequence. The vertical dotted lines in the bottom panel indicate the exon-intron junction, and the red arrows indicate the mutation sites in the *Myo6* cDNA and genomic DNA.

Open-field behavioral testing revealed shaker/waltzer behavior, such as circling and hyperactive behavior, in *ksv*/*ksv* homozygous mice (Fig 1A and 1B). We confirmed that the shaker/waltzer behavior of *ksv*/*ksv* mice was caused by stereociliary defects in vestibular hair cells, on the basis of comparison of SEM images between the +/+ and *ksv/ksv* mice (Fig 1C and 1D). Degenerated and decreased bundles were observed in most stereocilia in the *ksv/ksv* mice. The elongation of stereociliary bundles in the lateral edge was also detected (Fig 1D). Moreover, the bases of stereocilia are usually tapered in +/+ mice (Fig 1C’), but the taper region of several stereocilia was absent in *ksv*/*ksv* mice (Fig 1D’). In addition, the bases of the stereocilia exhibited bulging. The profound hearing loss of the *ksv*/*ksv* mutants was confirmed by detection of ABR. The *ksv*/*ksv* mice had no discernable wave forms at any of the amplitudes tested (Fig 2A). The observation of the stereocilia in the inner hair cells (IHCs) and outer hair cells (OHCs) in the organ of Corti in the *ksv* mice indicated that all of the deaf shaker/waltzer mice showed stereociliary defects. Fig 2B/B’ and 2C/C’ show SEM images of the stereocilia in +/+ and *ksv/ksv* homozygous mice, respectively. Several severely defective phenotypes of stereocilia, such as loss of tapers, bulged bases, bulbous tips and gigantic bundles, which are the predicted results of stereociliary fusion, were observed in the mature stereocilia of *ksv/ksv* mice (Fig 2C and 2C’). Although previous studies have reported that mice with mutations in any of several deafness-related genes show stereociliary fusion, the typical phenotypes of the hair cells from the organ of Corti in *ksv* mice were very similar to the early postnatal and mature stereocilia phenotypes of *Myo6* mutants, including *Myo6*^*sv*^ mice [23, 34] and the allele series *Myo6*^-^ [20] and Charlie (*Myo6*^*chl*^) [35]. Therefore, we generated (JF1 × B6J-*ksv/ksv*) F_1_ × B6J-*ksv/ksv* and (MSM × B6J-*ksv/ksv*) F_1_ × B6J-*ksv/ksv* backcrossed progeny and genotyped the animals by using a polymorphic marker on chromosome 9, including a marker within *Myo6*, to ensure that the phenotypes of the progeny correlated with their genotypes. The analysis revealed a linkage association with an approximately 24 Mb region that included *Myo6* (Fig 3A). Moreover, +/*ksv* heterozygous mice were crossed to the *Myo6*-null (*Myo6*^-/-^) mutant [20] mice to perform an allelism test. This mating yielded 15 offspring, of which seven showed shaker/waltzer behavior, thus demonstrating that the *ksv* mutation was allelic with *Myo6*^-^. Next, we sequenced the open reading frame of *Myo6* amplified from cDNA of the +/+ (*Myo6*^+/+^), +/*ksv* heterozygous and *ksv/ksv* homozygous mice and identified a c.1381G>A mutation in the *ksv* mice (Fig 3B, top) (DDBJ/EMBL/GenBank accession number: LC158863). The c.1381G>A mutation is a missense mutation that changes a glutamic acid residue at position 461 of the MYO6 protein to a lysine residue (p.E461K). Moreover, the c.1381G>A mutation was identified at the last position of exon 12 of *Myo6*, one base before the splice-donor site (Fig 3B, bottom). No other mutations have been discovered in the genomic region of *Myo6*, including all coding exons, a non-coding exon, UTRs and the *Myo6* promoter (approximately 350-bp upstream of non-coding exon 1) [36]. Together, these results strongly suggested that the congenital vestibular dysfunction, profound hearing loss and stereociliary fusion observed in *ksv*/*ksv* mice were caused by the c.1381G>A mutation in *Myo6*.

### The *ksv* mutation of *Myo6* causes alternative splicing errors leading to decreased MYO6 protein levels

**Fig 4:**
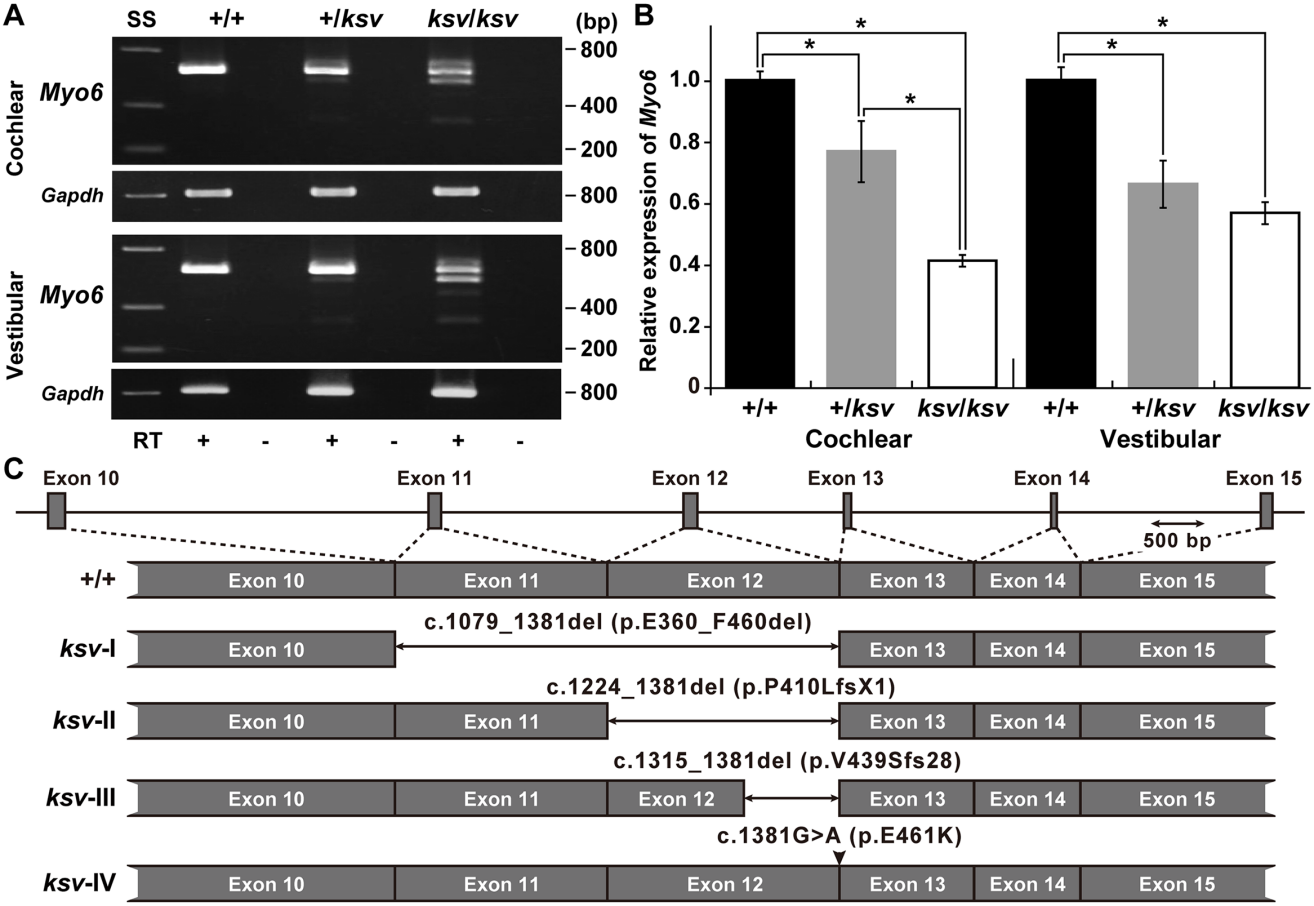
Alternative splicing errors and decrease in *Myo6* mRNA caused by the *ksv* mutation. A. RT-PCR analysis of *Myo6* expression in cochlear and vestibular RNA from +/+, +/*ksv* and *ksv/ksv* mice at P30. The upper panels show the RT-PCR products obtained from both tissues by using *Myo6*-specific primers located in exons 10 and 15/16 (S1 Table). The integrity of the cDNA was confirmed by using a *Gapdh* control (bottom panels). B. Relative levels of *Myo6* mRNA in the cochlear and vestibular tissues of +/+ (*n* = 3), +/*ksv* (*n* = 3) and *ksv/ksv* (*n* = 3) mice at P30. *P < 0.05. C. Patterns of exon skipping in *Myo6* alternatively spliced isoforms transcribed in the *ksv* mutant. The two-direction arrows and arrowhead indicate deletions and the c.1381G>A missense mutation, respectively.

To investigate the effects of the *ksv* mutation on the expression of *Myo6* transcripts, we performed RT-PCR analysis using cochlear and vestibular RNA from +/+, +/*ksv* heterozygous and *ksv/ksv* homozygous mice. We detected multiple bands in both the cochlear and vestibular RNA of +/*ksv* and *ksv/ksv* mice (Fig 4A), thus indicating that abnormal alternatively spliced isoforms were produced by the *ksv* mutation one base before the splice-donor site of exon 12. Next, to quantify differences in the expression levels of the gene in these mice, we performed qRT-PCR using cochlear and vestibular RNA from +/+, +/*ksv* and *ksv/ksv* mice. Both cochlear and vestibular *Myo6* RNA were present in significantly lower amounts in *ksv* mutant mice than in +/+ mice. The relative abundance of *Myo6* transcripts in the cochlear RNA of +/*ksv* and *ksv/ksv* mice was approximately 77.3 and 41.4%, respectively, that of the wild-type levels (Fig 4B). Although the differences in vestibular RNA levels between the +/*ksv* and *ksv/ksv* mice were not statistically significant, similar gradual decreases in the vestibular *Myo6* RNA were detected in +/*ksv* (66.7%) and *ksv/ksv* (57.2%) mice (Fig 4B). Multiple bands were characterized by the DNA sequencing of the PCR products. The results suggested that at least four alternatively spliced isoforms (*ksv*-I–IV, Fig 4C) were transcribed in the cochlear and vestibular tissues of *ksv* mutants. The *ksv*-I, II and III isoforms entirely lacked exons 11 and 12 (c.1079_1381del), entirely lacked exon 12 (c.1224_1381del) and contained a partial sequence of exon 12 (c.1315_1381del), respectively. The predicted translation product of the *ksv*-I isoform featured a 101-aa in-frame deletion (p.G360_F460del) within the motor domain of the protein. The complete/partial lack of exon 12 in the *ksv*-II and III isoforms was predicted to lead to a frameshift that truncates the peptide chain because of the generation of a stop codon at amino acid positions 411 (p.P410LfsX1) and 467 (p.V439SfsX28), respectively. The normally spliced *ksv*-IV isoform included the p.E461K mutation.

**Fig 5:**
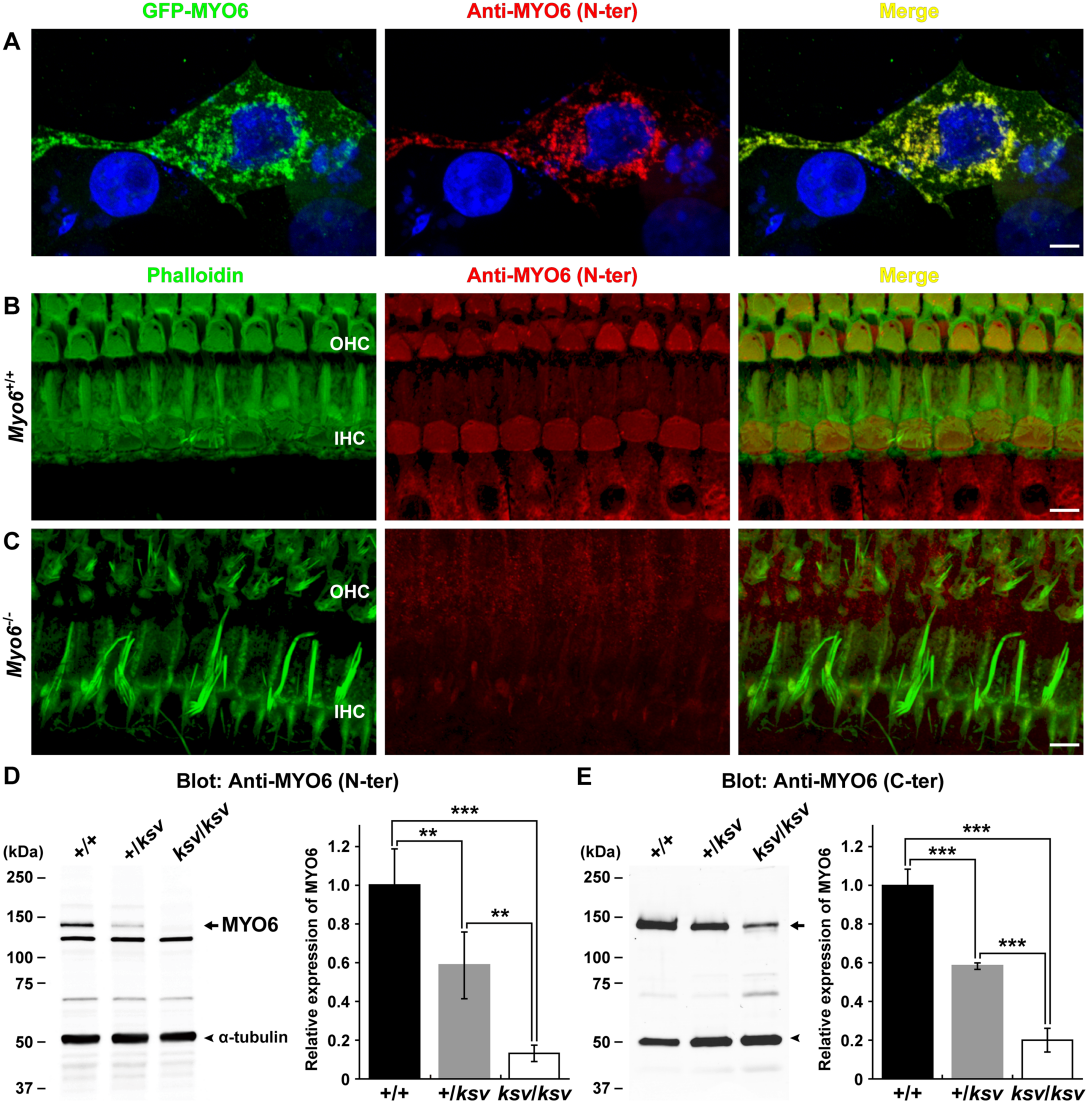
Decreased expression levels of the MYO6 protein in *ksv* mice. A–C. Characterization of a novel anti-MYO6 (N-ter) antibody against a peptide in the N-terminal motor domain of the MYO6 protein. A. Evaluation of the specificity of the antibody in COS7 cells transiently transfected with GFP-MYO6. COS7 cells transfected with GFP-MYO6 (green) and stained with the new antibody (red) are shown. Green and red signals are co-localized in the perinuclear region. Scale bar = 5 μm. B and C. Expression analysis of MYO6 protein in *Myo6*^+/+^ (B) and *Myo6*^-/—^null mutant (C) mice using the anti-MYO6 (N-ter) antibody. Whole-mount immunostaining of the cochlear hair cells from *Myo6*^+/+^ (B) and *Myo6*^-/-^ (C) mice double-labeled with phalloidin (green) and the antibody (red). The right panels of B and C show merged images after double-labeling with phalloidin and anti-MYO6 (N-ter) antibody. Expression of MYO6 is completely ablated in the hair cells of *Myo6*^-/-^ mice (C). Scale bar = 5 μm. D and E. Western blot analysis of homogenates prepared from the inner ears of +/+, +/*ksv* and *ksv/ksv* mice at P30 detected with anti-MYO6 (N-ter, D) and MYO6 (C-ter, E) antibodies (S2 Table). Note the stepwise decrease in the intensity of the MYO6-specific band at approximately 145 kDa (arrows), which is recognized by both antibodies in homogenates from +/*ksv* and *ksv/ksv* mice. The samples were processed for indirect immunofluorescence using an anti-α-tubulin antibody (arrowheads). The right graphs show the densitometric quantification of the MYO6 expression levels detected by western blot analysis. The values shown in each graph indicate the mean relative expression levels and the SDs in triplicate. **P < 0.01; ***P < 0.001.

To confirm the decreased expression of the MYO6 protein and to detect the presence of the truncated MYO6 protein in *ksv* mutants, we generated an antibody, anti-MYO6 (N-ter), to a peptide within the motor domain in the N-terminal region of the *ksv* mutation. The specificity of the antibody was validated by immunocytochemistry with GFP-tagged MYO6-transfected COS7 cells. The anti-MYO6 (N-ter) antibody showed specificity in recognizing the GFP-tagged MYO6 in transfected cells, and there was no background staining in nontransfected cells (Fig 5A). The specificity of the antibody was also validated by immunohistochemistry, which showed the typical localization of MYO6 in the cuticular plates and cytoplasm of cochlear hair cells in *Myo6*^+/+^ mice (Fig 5B), and by a lack of labeling of hair cells from the *Myo6*^-/-^ homozygous mutant (Fig 5C). In an immunoblot analysis using the anti-MYO6 (N-ter) antibody, the bands at approximately 145-kDa that have previously been reported to be the major bands of MYO6 [18, 20, 30] recognized by this antibody were detected in inner ear extracts from +/+ (*n* = 3), +/*ksv* (*n* = 3) heterozygous and *ksv/ksv* (*n* = 3) homozygous mice, although several additional bands were detected in immunoblots of inner ear extracts (Fig 5D, left). The intensity of the 145-kDa bands was markedly decreased in the +/*ksv* and *ksv/ksv* mice (Fig 5D, left), being 1.71 and 7.59 times less abundant in the inner ears of +/*ksv* and *ksv/ksv* mice, respectively, than in those of +/+ mice (Fig 5D, right). We also performed an immunoblot analysis using a rabbit polyclonal antibody, anti-MYO6 (C-ter), which was raised against a recombinant polypeptide with the same sequence as a portion of the C-terminal cargo-binding domain of MYO6 (S2 Table) [18, 30]. In an immunoblot analysis using the anti-MYO6 (N-ter) antibody, a faint signal of the approximately 145-kDa band was detected in the extract of *ksv*/*ksv* mice (Fig 5E, left). The stepwise decrease in the expression of MYO6 in the inner ears of +/*ksv* and *ksv/ksv* mice was determined by quantification with the anti-MYO6 (C-ter) antibody (Fig 5E, right). The above results showed that the levels of *Myo6* RNA and MYO6 protein were decreased in *ksv* mutants, probably because of the degradation of at least the *ksv*-II (p.P410LfsX1) and -III (p.V439SfsX28) isoforms in *ksv* mice by the nonsense-mediated mRNA decay (NMD) pathway [37].

### Hair cells of *ksv* mutants show decreased expression levels and mislocalization of MYO6

**Fig 6:**
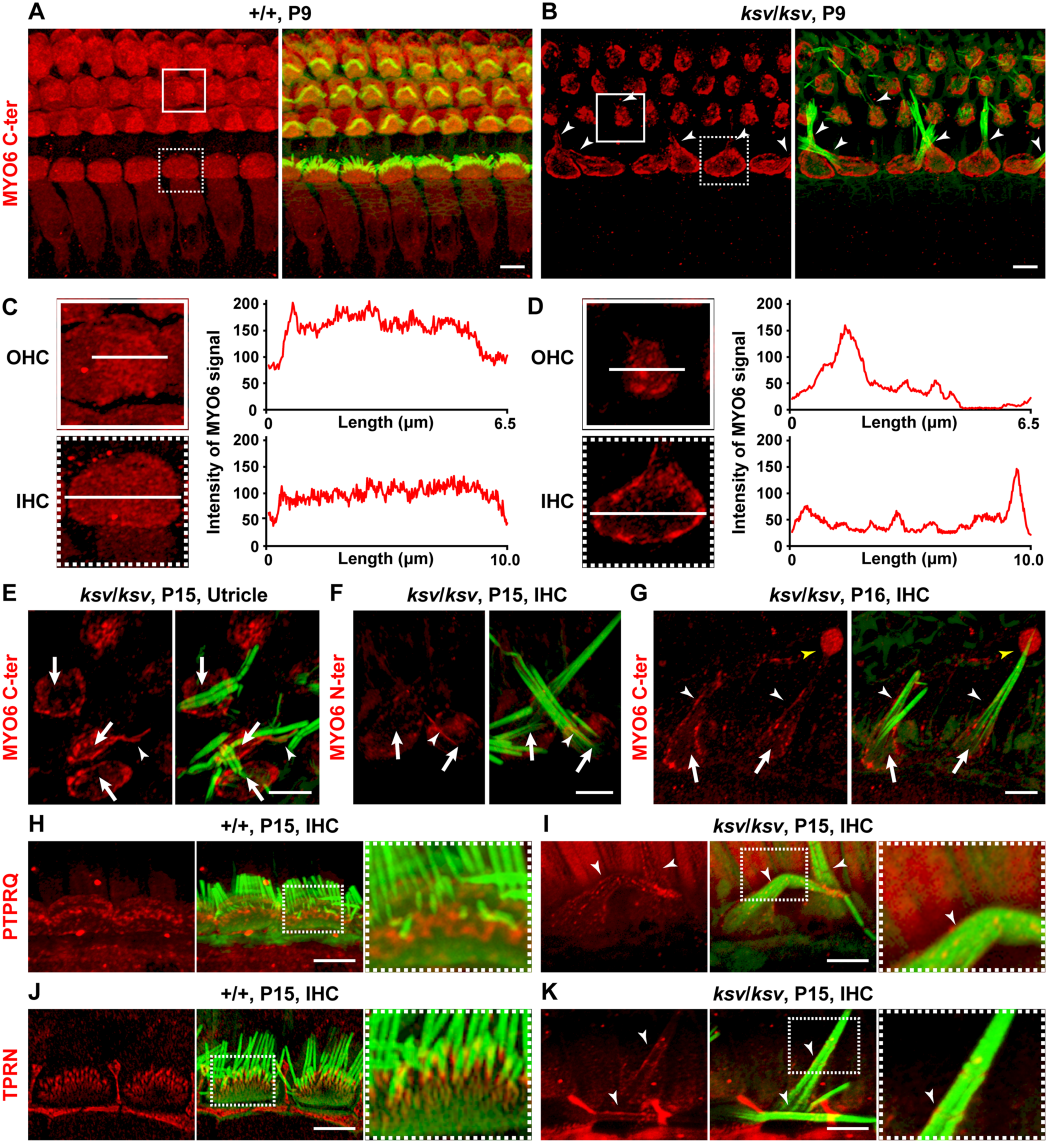
Decreased expression levels and/or mislocalization of MYO6 and stereociliary taper-specific proteins in the hair cells of *ksv*/*ksv* mice. A–G. Double staining for MYO6 (red: C-ter, A–E and G; N-ter, F) and phalloidin (green) in the cochlear (A–D, F and G) and vestibular (E) hair cells of +/+ (A and C) and *ksv/ksv* homozygous (B and D–G) mice. MYO6 immunoreactivity was detected in the cuticular plates, pericuticular necklaces, and cytoplasm of the OHCs and IHCs of +/+ mice but was not observed in the stereocilia (A). In *ksv/ksv* mice (B and D–G), MYO6 signals were not observed in the cytoplasm (arrows) but were observed along the length (arrowheads) of the stereocilia in the cochlea (B, F and G). Highly magnified images of representative expression patterns of the OHCs (boxes) and IHCs (dotted boxes) in A and B are shown in the left panels of C and D. The intensity of the white lines in the left panels indicates the degree of decrease in the MYO6 signals in the cuticular plates in *ksv/ksv* mice. A similar decrease in the cuticular plates was observed in the utricle hair cells of *ksv/ksv* mice (E) and in IHCs stained with the anti-MYO6 (N-ter) antibody (F). A bulbous signal (yellow arrowhead) of MYO6 was detected at the tips of the fused stereocilia in *ksv/ksv* mice (G). H–K. Double staining for stereociliary taper-specific proteins (red: PTPRQ, H and I; TPRN, J and K) with phalloidin (green) in the IHCs of +/+ (H and J) and *ksv/ksv* (I and K) mice. Highly magnified images of dotted boxes in the middle panels of H–K are shown in each right panel. Arrowheads indicate the mislocalization along the length of the stereocilia of each protein in *ksv*/*ksv* mice (I and K). Scale bars = 5 μm.

To determine the expression of MYO6 proteins within the inner ear hair cells of *ksv*/*ksv* mice, we performed immunohistochemistry using anti-MYO6 antibodies. Fig 6A and 6B shows the pattern of expression of MYO6 in the hair cells of the organ of Corti at P9 in +/+ and *ksv*/*ksv* mice. Strong MYO6 signals were detected in the cuticular plate and cytoplasm in both IHCs and OHCs. In *ksv*/*ksv* mice, MYO6 signals were detected in both IHCs and OHCs. However, the signals were completely lost from the cytoplasm and decreased in the cuticular plates of the hair cells in *ksv*/*ksv* mice. We compared the intensities of the MYO6 signals on the surfaces of the hair cells in +/+ and *ksv*/*ksv* mice. The intensities were fairly uniform in the +/+ mice (Fig 6C), whereas in the *ksv*/*ksv* mice, the intensity of the immunostaining was decreased in the cuticular plate, except for the pericuticular necklace, in both IHCs and OHCs (Fig 6D). Moreover, we found mislocalization of the MYO6 signals along the length of the stereocilia in *ksv*/*ksv* mice (Fig 6B). The mislocalized signals in *ksv*/*ksv* mice appeared to be distributed toward the basal region of the stereocilia. Similar decreases in the MYO6 signals in the cuticular plates and abnormal localization of the MYO6 signals in the stereocilia were observed in the vestibular hair cells (Fig 6E) and by immunostaining using the anti-MYO6 (N-ter) antibody (Fig 6F). Although the MYO6 expression profiles of the hair cells in *ksv*/*ksv* mice scarcely changed as the hair cells matured, the signals gradually became evenly distributed along the length of the stereocilia (Fig 6G). Moreover, we found bulbous MYO6 signals at the tips of a minor population of stereocilia in *ksv*/*ksv* mice (Fig 6G).

The mislocalization along the length of the stereocilia is suggested in the expression of the *ksv*-I and -IV mutant isoforms, which may include the mutations p.G360_F460del and p.E461K, respectively (Fig 4C). To assess the functional effects of these in-frame and missense mutations in the myosin motor domain on the expression and localization of MYO6, we examined the subcellular expression of the mutant MYO6 fused to a fluorescently tagged protein and compared it with the localization of the MYO6 fusion protein in COS7 cells. In transiently transfected cells producing wild-type full-length GFP- and DsRed-MYO6, the signals were detected in the perinuclear region, similarly to the previously reported localization in mammalian cells [21, 30, 38] (Fig 5A, S1A and S1D Fig). The localization of the p.G360_F460del and p.E461K mutant constructs was similar to that of the wild-type protein in single transfection cells (S1A–S1F Fig). In addition, most of the signals of the wild-type and mutant proteins were merged in the co-transfected cells (S1G and S1H Fig). Although we performed the co-transfections by using smaller tagged (FLAG-tagged) proteins, it is difficult to explain the difference in the localization between the wild-type and mutant proteins (S1I and S1J Fig). Thus, these *in vitro* experiments did not reveal how the p.G360_F460del and p.E461K mutations affect the motor function of the MYO6 protein.

Next, we studied the localization of PTPRQ and TPRN in the hair cells of *ksv*/*ksv* mice because these proteins may form a complex with MYO6 at the base of the stereociliary taper and/or the surface of the cuticular plate membrane [21, 22]. In +/+ mice, the expression of both PTPRQ and TPRN was restricted to the bases of the stereocilia (Fig 6H and 6J), as previously shown [9, 12, 15, 21, 22]. Earlier studies have also demonstrated that the localization of the proteins expressed in the stereociliary base is disrupted in MYO6-null *Myo6*^*sv*/*sv*^ homozygous mutants, and these proteins are instead distributed throughout the entire length of the fused stereocilia [21, 22]. We observed similar changes in the localization of both PTPRQ and TPRN, from localization at the bases of the stereocilia in +/+ mice to distribution along their entire length in *ksv*/*ksv* mice (Fig 6I and 6K).

### Stereociliary fusion of *ksv* mutants accompanies deformation of cuticular plate membranes

**Fig 7:**
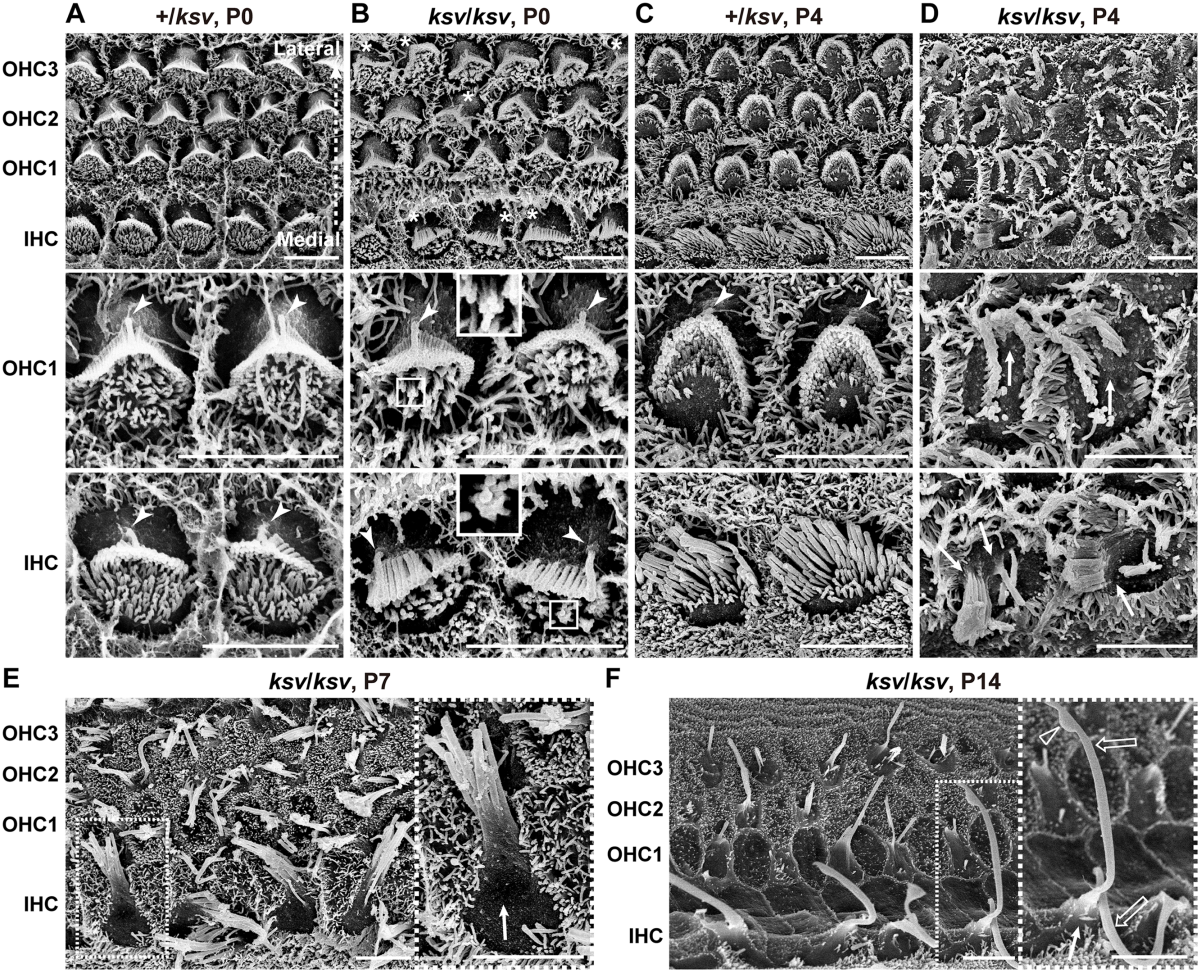
Rapidly progressive stereociliary fusion of the cochlear hair cells in *ksv*/*ksv* mice during early postnatal stages. A–D. Comparison of stereociliary phenotypes between the +/*ksv* heterozygous (A and C) and *ksv*/*ksv* homozygous (B and D) mice at P0 (A and B) and P4 (C and D). SEM images showing stereocilia in the hair cells from the middle area of the cochlea. Highly magnified images of the OHCs (middle panels) and IHCs (bottom panels) are shown. The arrowheads and arrows indicate the kinocilia and bulging basal region of the stereocilia, respectively. The bundling of the stereocilia in both the OHCs and IHCs of *ksv*/*ksv* mice at P0 are magnified (white boxes). E. Stereociliary phenotypes in the hair cells from the middle area of the cochlea in *ksv*/*ksv* mouse at P7. The highly magnified image (right panel) in the white dotted box in the left panel shows the bulging basal region (arrow) in the fused stereocilia in IHCs. F. Stereociliary phenotypes in the hair cells from the apex area of the cochlea in a *ksv*/*ksv* mouse at P14. The highly magnified image (right panel) in the white dotted box in the left panel shows the bulging basal region (arrow) and bulbous tip (open arrowhead) in the elongated giant stereocilia (open arrows) of the IHCs. Scale bars = 5 μm.

The MYO6 expression of the *ksv* mutants was appreciably decreased in the cuticular plates and cytoplasm and was mislocalized along the length of the stereocilia (Fig 6A–6G). Therefore, we next observed stereocilia phenotypes from the cochlear hair cells in *ksv*/*ksv* mice at an early developmental stage to determine the *ksv* mutant-specific phenotypes and generate new findings related to stereociliary fusion in *Myo6* mutants. Fig 7A and 7B show SEM images of the surfaces of the OHCs and IHCs of +/*ksv* heterozygous and *ksv*/*ksv* homozygous mice at P0. This stage is when the rank formation of the stereocilia occurs [39]. In both +/*ksv* and *ksv*/*ksv* mice, the short population of stereocilia covered the lateral edges from the medial edges of the cuticular plates, and the tall population was shaped and ranked at the lateral edges of both the OHCs and IHCs with single kinocilia (Fig 7A and 7B). Although the stereocilia of +/*ksv* mice developed normally, similarly to those of +/+ mice at the same stage [40] (Fig 7A), the stereocilia of *ksv*/*ksv* mice showed abnormal formation, such as misorientation in several hair cells and the bundling of adjacent stereocilia in the short population (Fig 7B). Mislocalization of the kinocilia was also detected in several hair cells in *ksv*/*ksv* mice (Fig 7B). We quantified the kinocilia positions on the hair cells of the organ of Corti in +/+ and *ksv*/*ksv* mice according to a previous study [41, 42]. In the *ksv*/*ksv* mice, mislocalization of the kinocilia was detected in IHCs and OHCs at prevalence of 45.5 and 50.9%, respectively (S2 Fig).

The rank formations of the stereocilia were more advanced in the hair cells of +/*ksv* mice at P4. The stereocilia largely migrated to the lateral edges of the cuticular plates and were differentiated into distinct ranks according to similar lengths (Fig 7C). Although the stereocilia of *ksv*/*ksv* mice at P4 were differentiated at the edges on the cuticular plates, the locations were disordered (Fig 7D). Moreover, the stereocilia bundles were clearly thickened and their numbers decreased in the *ksv*/*ksv* mice, thus suggesting that the stereocilia of the *ksv*/*ksv* mice were already fused at this developmental stage. In addition, we found bulging of the cuticular plate membrane at the bases of the stereocilia at this stage (Fig 7D). At P7, the stereocilia were clearly fused and elongated in the *ksv*/*ksv* mice. The bulged cuticular plate membranes became clear, especially in the IHCs (Fig 7E). By P14, the stereocilia were completely fused in both the IHCs and OHCs of the *ksv*/*ksv* mice. Bulging tapers, bulbous tips and gigantic bundles were observed at this stage, as well as in adult *ksv*/*ksv* mice (Figs 2C, 2C’ and 7F).

**Fig 8:**
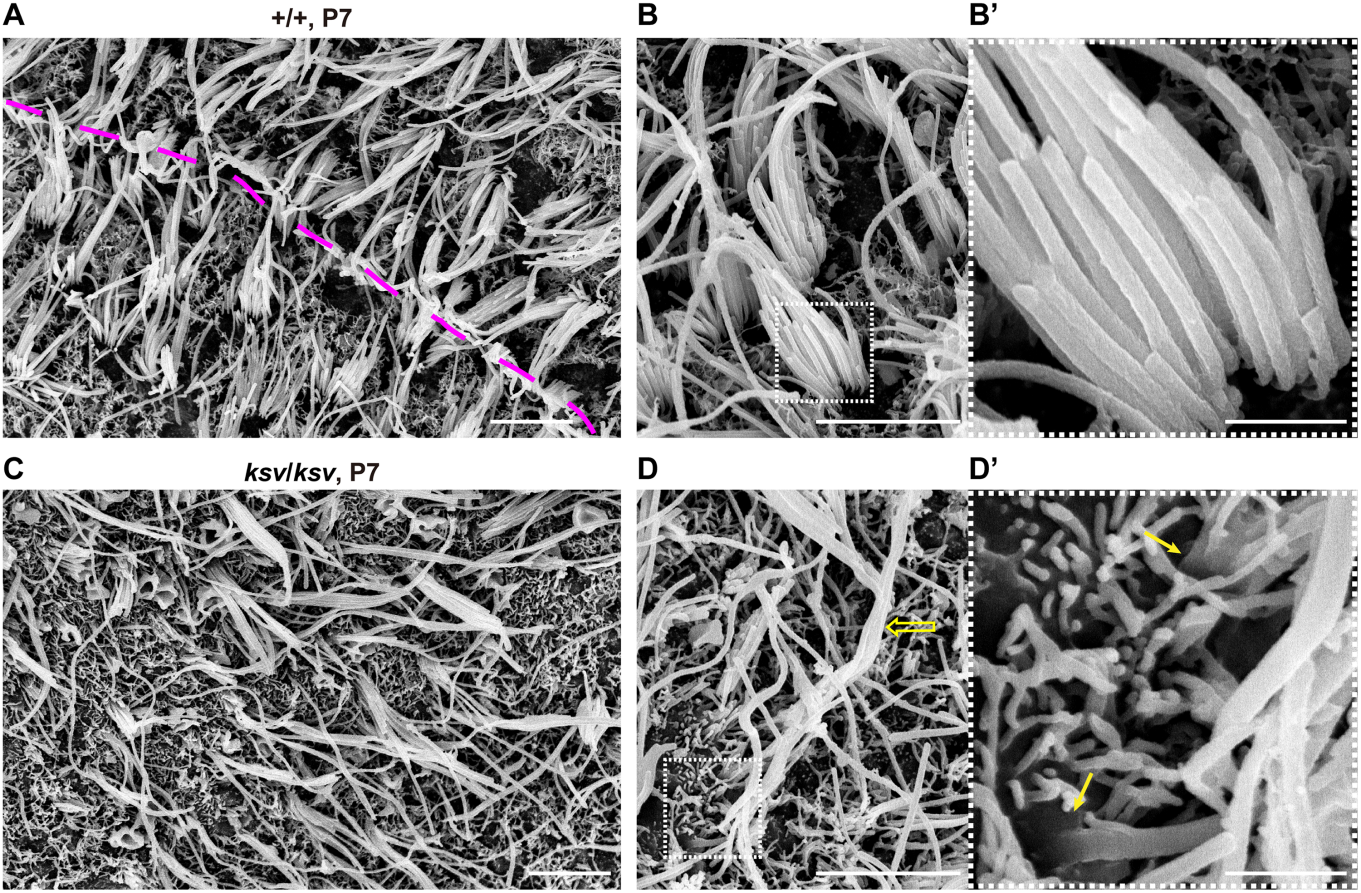
Misorientation and fusion at the bases of the stereocilia in vestibular hair cells in *ksv*/*ksv* mice. A–D SEM images of stereocilia in the utricles of the +/+ (A and B) and *ksv/ksv* (C and D) mice at P7. The dashed pink line indicates the virtual line of polarity reversal (LPR) in vestibular hair cells (A). The magnified images of stereociliary bundles from +/+ and *ksv*/*ksv* mice are shown in B and D. The elongated stereociliary bundle (open arrow) is noted in the utricle. The highly magnified images in the white dotted boxes of B and D are shown in the right panels. Arrows indicate a slightly bulged base and a loss of taper in stereocilia in *ksv*/*ksv* mice. Scale bars = 5 μm (A, B, C and D), and 1 μm (B’ and D’).

Next, we investigated the phenotypes of the stereocilia from vestibular hair cells and whether similar phenotypes were present in those of *ksv*/*ksv* mice. Fig 8A shows an SEM image of the utricle hair cells in +/+ mice at P7. The stereociliary bundles are polarized and are oriented either uniformly or in opposite directions across a line of polarity reversal (LPR) in utricle hair cells [40, 43]. However, orientations of the bundles in *ksv*/*ksv* mice were irregulars, and it was difficult to identify the LPR in the utricle (Fig 8C). Moreover, the stereociliary bundles were clearly abnormal at this stage. The rank formations of the most stereocilia were vague in *ksv*/*ksv* mice compared with +/+ mice (Fig 8A–8D). The elongated stereocilia were detected in *ksv*/*ksv* mice. Although the bases of the stereocilia were already tapered in +/+ mice at this stage (Fig 8B’), the tapers were lost, probably because of stereociliary fusion and/or bulging of the cuticular plate in *ksv*/*ksv* mice (Fig 8D’).

**Fig 9:**
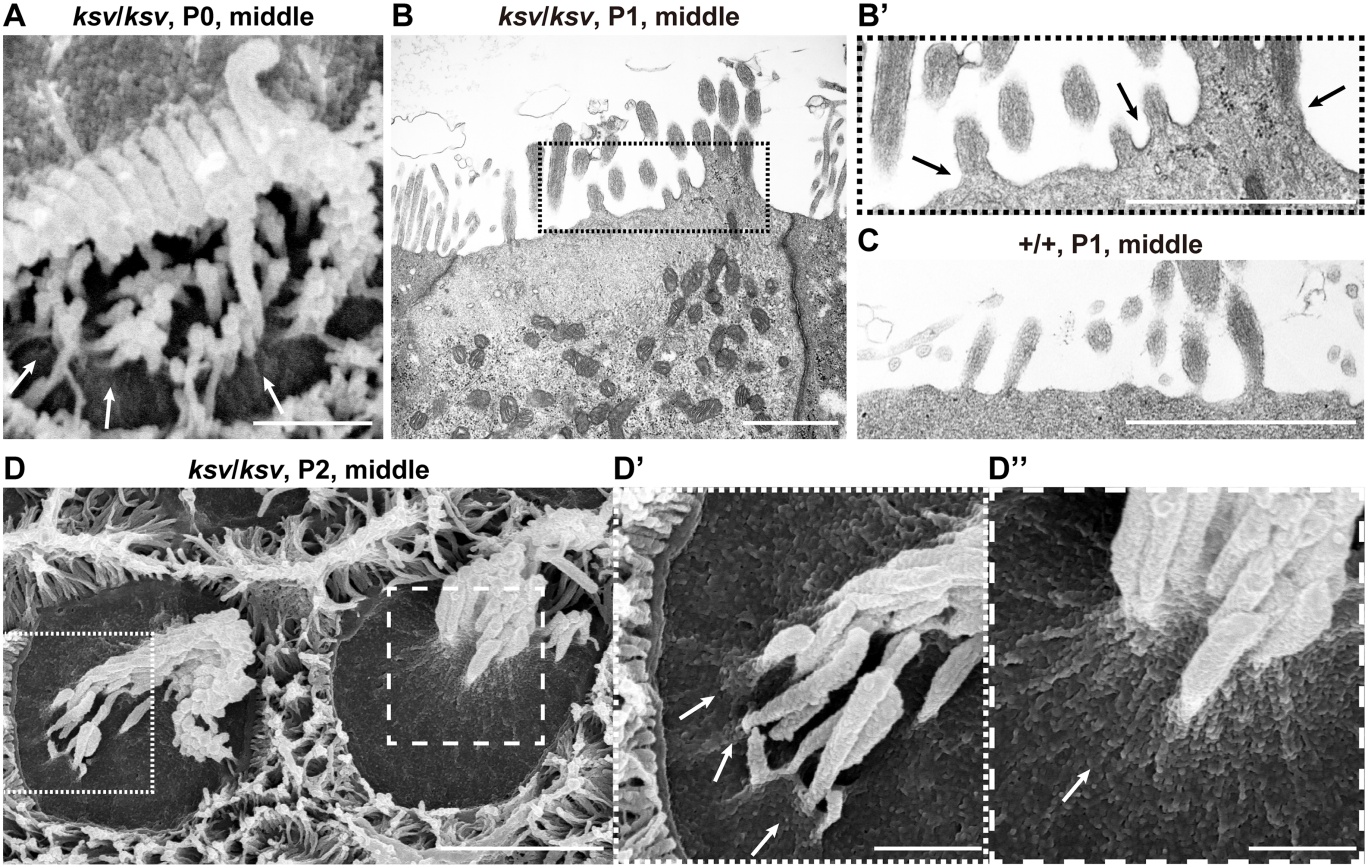
Deformation of the cuticular plate membranes of IHCs of *ksv*/*ksv* mice during the process of stereociliary fusion. A. SEM image showing stereocilia in IHCs from the middle area of the cochlea in a *ksv*/*ksv* mouse at P0. Raised membrane of the cuticular plate (arrows). B. TEM image showing apical regions of the IHCs of a *ksv*/*ksv* mouse at P1. Highly magnified image of the stereociliary base shown in B’. C. Highly magnified TEM image showing the stereociliary base of the IHCs of a +/+ mouse at P1. D. Typical phenotypes of the apical surfaces of IHCs of *ksv/ksv* mice at P2. Highly magnified images in dotted and dashed boxes of D show the bulging of the cuticular plate membrane in D’ and D” (arrows), respectively. Scale bars = 3 μm (D), and 1 μm (A–C, D’ and D”).

**Fig 10:**
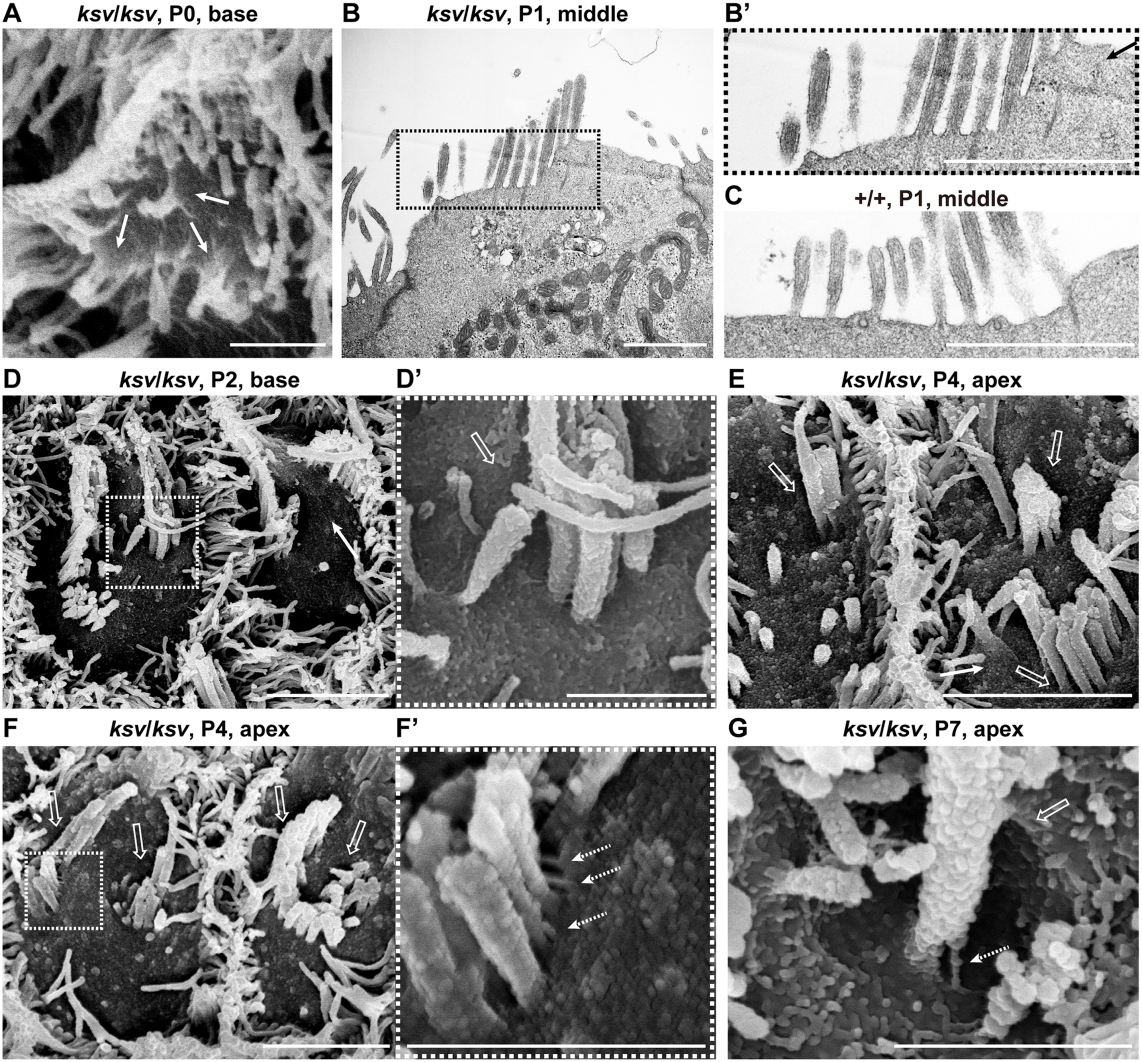
Deformation of the cuticular plate membranes of OHCs of *ksv*/*ksv* mice during the process of stereociliary fusion. A. SEM image showing the raised stereociliary bases in the OHCs from the base area of the cochlea in a *ksv*/*ksv* mouse at P0. Arrow indicates the raised membrane of the cuticular plate. B. TEM image showing the apical regions of OHCs from the middle area of the cochlea in a *ksv*/*ksv* mouse at P1. Highly magnified image of the stereociliary base shown in B’. C. Highly magnified TEM image showing the stereociliary base in OHCs of a +/+ mouse at P1. D. Typical phenotypes of the apical surfaces of the OHCs from the base area of the cochlea in *ksv/ksv* mice at P2. Highly magnified image (D’) in the dotted box of D shows the subsidence (open arrow) of the cuticular plate membrane in the stereociliary base. E–G. Phenotypes of the apical surfaces of the OHCs from the apex area of the cochlea in *ksv/ksv* mice at P4 (E and F) and P7 (G). Open arrows indicate the pocket-like structures of the cuticular plate detected in the bases of the incorporated stereociliary bundles. Highly magnified image (F’) in the dotted box of F shows the connections via links (dotted arrows) between the stereocilia and the cuticular plate membrane detected in the OHCs. Scale bars = 3 μm (D, E and F), and 1 μm (A–C, D’, F’ and G).

In the phenotypic analysis of the stereocilia during early postnatal development, we observed several typical phenotypes in the process of stereociliary fusion in cochlear and vestibular hair cells of *ksv*/*ksv* mice (Figs 1D’, 2C’, 7D–7F and 8D’). To investigate the more detailed phenotypes in the process of stereocilary fusion in *ksv*/*ksv* mice, we analyzed the highly magnified electron microscopic images of stereocilia at early postnatal stages. This analysis focused on stereocilia from the cochlear hair cells, because the phenotypes at early postnatal stages were more severe than those of vestibular hair cells and could be relatively easily observed. We observed raised and bulged cuticular plate membranes at the bases of the stereocilia in cochlear hair cells of *ksv*/*ksv* mice at P4–14 (Fig 7D–7F). This phenotype was observed in several IHCs from the base area to the middle of the cochlea at an earlier developmental stage, P0 (Fig 9A). At P1 and P2, the bulging of the cuticular plates in the stereociliary base and the loss of taper were observed in most of the IHCs from the *ksv*/*ksv* mice (Fig 9B and 9D and S3 Fig). The phenotypes were classified into two patterns of slight bulging in either a single stereocilium or a few stereocilia (Fig 9B’ and 9D’) and severe bulging in the bases of the growing stereocilia (Fig 9B’ and 9D”). We also observed the bulging of the cuticular plate membranes in the stereociliary base in the OHCs of *ksv*/*ksv* mice at P0 (Fig 10A), although this phenotype was restricted to the base of the cochlea. At P1 and P2, severe bulging of the cuticular plate was detected in the growing stereocilia in OHCs (Fig 10B and S3 Fig), but several stereocilia were tapered at their bases, similarly to those of +/+ mice (Fig 10B’ and 10C). Interestingly, we found the other phenotype in which subsidence appeared on cuticular plate membranes and then was incorporated with the stereocilia bundles in several OHCs of *ksv*/*ksv* mice at P2 (Fig 10D and 10D’). The hollows in the cuticular plate became pocket-like structures at P4 and P7 (Fig 10E and 10F, S4A and S4B Fig), and the incorporation of stereocilia into the cuticular plates was detected in most of the OHCs from *ksv*/*ksv* mice (S4 Fig). Although the similar phenotype was observed in IHCs, it was less prevalent/common (S4A, S4A’ and S4C Fig). In addition, we discovered a connection via links between the incorporated stereociliary bundle and the cuticular plate membranes (Fig 10F’ and 10G).

### Stereociliary fusion of the *ksv* mutants accompanies disruption of the architecture at the apical surfaces of hair cells

**Fig 11:**
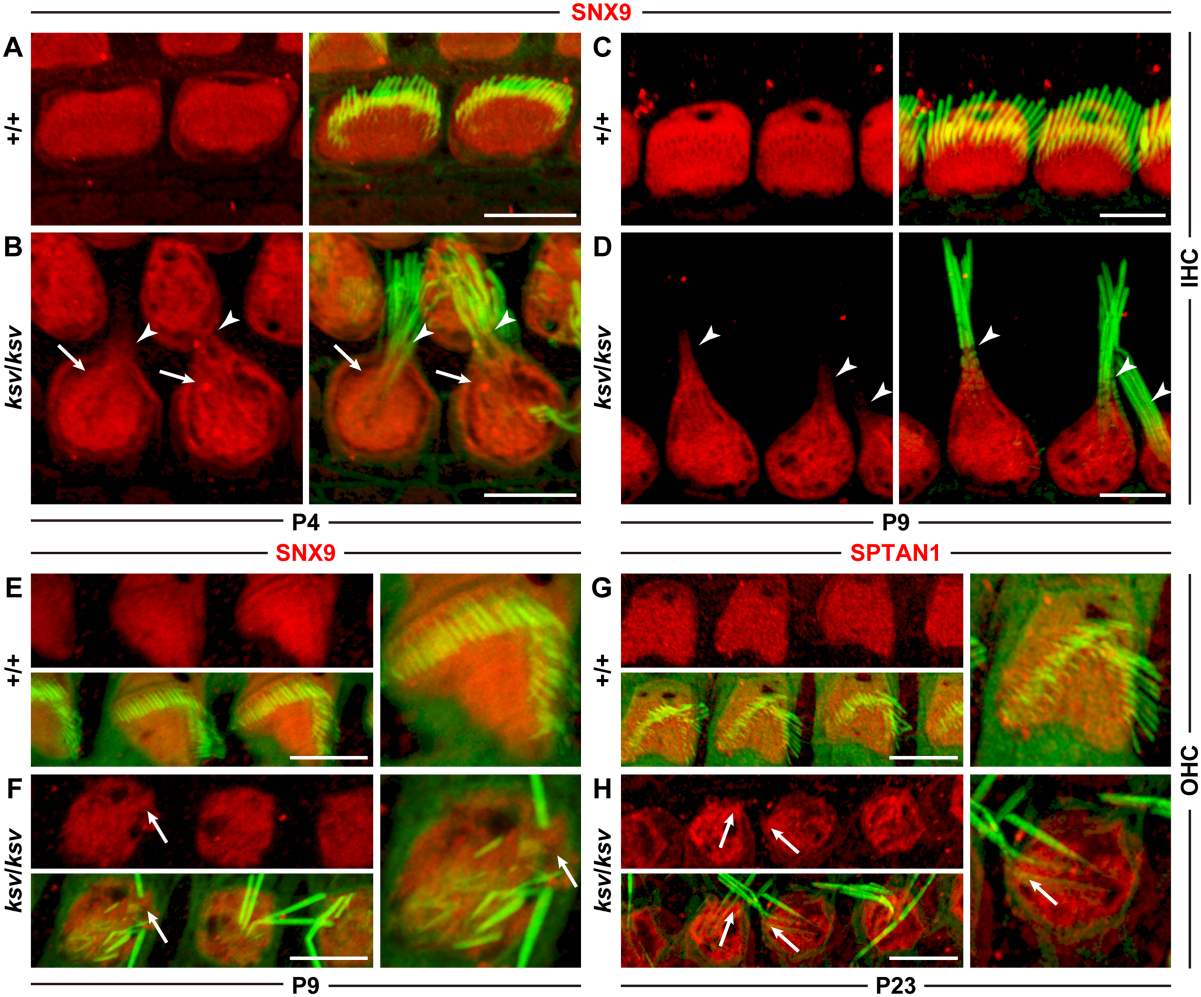
Signals of the cuticular plate markers migrate to the basal length of the stereocilia in IHCs of *ksv*/*ksv* mice. Localization of the markers for the cuticular plate, SNX9 (A–F) and SPTAN1 (G and H), in the IHCs (A–D) and OHCs (E–H) of +/+ (A, C, E and G) and *ksv*/*ksv* (B, D, F and H) mice at P4 (A and B), P9 (C–F) and P23 (G and H). In all images stained by each antibody, the left panels show the localization of their proteins (red), and the right panels show the merged image with phalloidin (green). Arrowheads and arrows indicate the red signals of the marker proteins detected in the stereocilia lengths and base, respectively. Scale bars = 5 μm.

We found deformations of the cuticular plate membranes, such as bulging and the subsidence of the cuticular plate membrane in the stereociliary base during the process of stereociliary fusion in the cochlear hair cells of *ksv*/*ksv* homozygous mice (Figs 7, 9 and 10). Moreover, we detected that the stereocilia bundles connected with the cuticular plate membranes through the links (Fig 10F’ and 10G). These phenotypes suggested that the stereocilia fuse with the cuticular plates through the deformation of the cuticular plate in the hair cells of *ksv*/*ksv* mice. To confirm this hypothesis, we performed an expression analysis of the marker proteins of the cuticular plates. We stained the hair cells by using antibodies to SNX9 and SPTAN1, which have previously been reported to be specific markers of the cuticular plate [29, 32]. Although we were not able to optimize the experimental conditions to prevent nonspecific binding when we used the SNX9 antibody for the staining of mature hair cells, the staining patterns confirmed that the signals of SNX9 and SPTAN1 were specifically localized to the cuticular plates of both the IHCs (Fig 11A and 11C) and OHCs (Fig 11E and 11G) of the +/+ mice. In the IHCs of the *ksv*/*ksv* mice, signals of the marker proteins were detected at the basal area of the fused stereociliary bundles at P4 (Fig 11B) and P9 (Fig 11D), thus indicating that the materials of the cuticular plates had migrated to the stereocilia. Although clear signals of the marker proteins were not detected in the stereocilia length of the immature and mature OHCs of the *ksv*/*ksv* mice, bulging was observed at the stereociliary base (Fig 11F and 11H).

**Fig 12:**
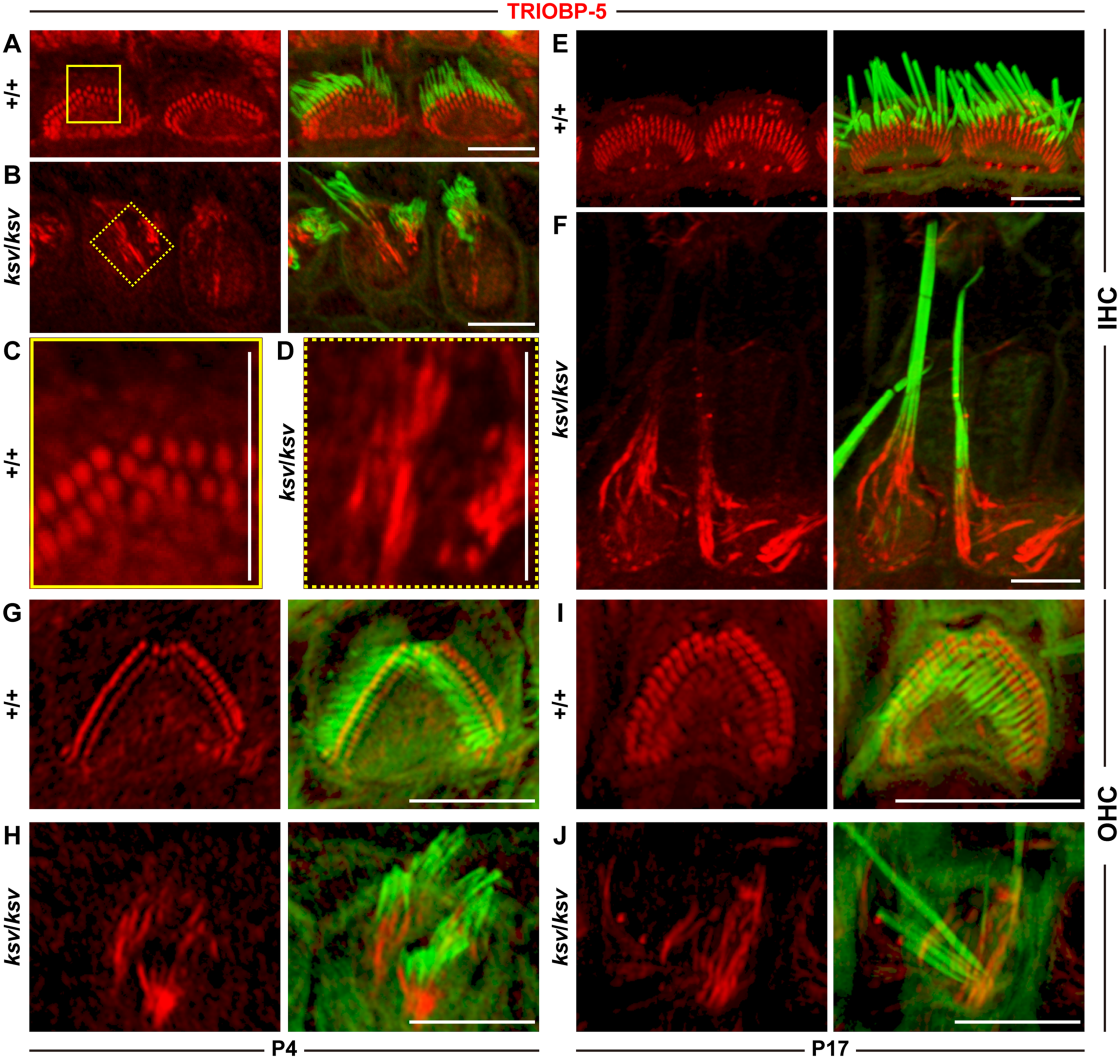
Elongation of the rootlets in the cochlear hair cells of the *ksv*/*ksv* mice. Localization of a marker for the rootlet, TRIOBP5, in the IHCs (A–F) and OHCs (G–J) of the +/+ (A, C, E, G and I) and *ksv*/*ksv* (B, D, F, H and J) mice at P4 (A–D, G and H) and P17 (E, F, I and J). The left and right panels show the localization of TRIOBP5 (red), and the right panels show the merged image with phalloidin (green). Highly magnified images of typical TRIOBP5 signals in IHCs from +/+ (A, yellow box) and *ksv*/*ksv* (yellow doted boxes) mice are shown in C and D, respectively. Scale bars = 5 μm.

Next, we stained the hair cells by using an antibody to TRIOBP-5, which recognizes an alternatively spliced isoform of TRIOBP and specifically labels the rootlets of the stereociliary bundles in mice [28]. At P4, we detected the signals of TRIOBP-5 in the rootlets of the stereociliary bundles in the IHCs of both +/+ (Fig 12A) and *ksv*/*ksv* (Fig 12B) mice. However, the signals of TRIOBP-5 were clearly extended in the *ksv*/*ksv* mice (Fig 12D), as compared with those of the +/+ mice (Fig 12C). By P17, the TRIOBP-5 signals appeared to have slightly extended in the IHCs of the +/+ mice, as compared with those at P4 (Fig 12E). In the IHCs of the *ksv*/*ksv* mice, the TRIOBP-5 signals were elongated toward the stereocilia tips, were significantly longer than those of the +/+ mice and were merged with the phalloidin signals representing the stained F-actin of stereocilia (Fig 12F). The TRIOBP-5 signals also appeared to extend toward the stereocilia tips in the OHCs of *ksv*/*ksv* mice at P4 and P17 (Fig 12G–12J), although the extension of this signal was unclear compared with those of the IHCs. Thus, our marker study suggested that the rootlets are elongated in the hair cells of *ksv*/*ksv* mice.

### Discussion

In this study, we identified a novel *Myo6* ^c.1381G>A^ mutation in *ksv* mice, on the basis of phenotypic similarities with existing *Myo6* mutants, genetic mapping and allelism tests, and characterized the balance and hearing phenotypes associated with this mutation. The *Myo6* ^c.1381G>A^ mutation causes a p.E461K change in the switch II loop of the protein (residues D456–F463) [16], an essential nucleotide-binding element in the motor domains of myosins [44–46]. In addition, the *Myo6* ^c.1381G>A^ mutation is located at the last position of exon 12 of *Myo6*, one base before the splice-donor site, and it leads to alternative splicing errors of the *Myo6* mRNA in *ksv* mutants. Owing to the splicing errors, most of the abnormal alternatively spliced isoforms of MYO6 are degraded (Fig 5D and 5E), thus leading to large decreases in the amount of MYO6 protein in the hair cells (Fig 6B and 6D–6G). The *ksv/ksv* homozygous mice displayed shaker/waltzer behavior and profound hearing loss caused by stereociliary fusion; these phenotypes are consistent with those of mice carrying the *Myo6*^*sv*/*sv*^ [6, 23, 34], *Myo6*^-/-^ [20] and *Myo6*^*chl*/*chl*^ [35] homozygous mutations. These homozygous *Myo6* mutants show a complete loss [6, 20] or significant decrease [35] in MYO6 in the inner ear hair cells. Although follow-up investigations including functional analyses to assess the function of the mutant MYO6 (p.E461K) protein, such as a kinetic analysis and a detailed phenotypic characterization of the +/*ksv* heterozygous mice, must be performed because the mutant MYO6 is expressed in the stereocilia and pericuticular necklace of the cochlear hair cells in *ksv* mice, we found that the stereociliary fusion in the *ksv*/*ksv* homozygous mutants caused large decreases in the amount of MYO6 in the hair cells.

As described in the Introduction, the results of previous studies have suggested that MYO6 moves toward the minus end of the actin filaments located near the rootlets of the stereocilia [47], because it transports cargo, such as PTPRQ, RDX and CLIC5, to the stereociliary taper region and/or the apical cuticular plate membrane. If MYO6 is absent from the hair cells, these tapering proteins broadly localized along the length of the stereocilia [21, 22]. Deletion of PTPRQ, RDX and CLIC5 results in stereociliary fusion in mice [8, 9, 21, 22, 26]. These data strongly suggest the presence of a MYO6/tapering protein complex that contributes to maintaining the architecture of the base of the stereocilium. Our immunohistochemical studies also showed an abnormal distribution of tapering proteins, PTPRQ and TPRN, throughout the stereocilia in the *ksv*/*ksv* homozygous mice, in which there was a massive decrease of MYO6 in the hair cells (Fig 6I and 6K). In the *ksv*/*ksv* mice, significant decreases in MYO6 were observed in the cuticular plates and cytoplasm of the hair cells (Fig 6B and 6D–6G). Therefore, the proper expression of MYO6 in the hair cell body (probably at the apical region) may require the recruitment of the tapering proteins to constrict the bases of the stereocilia.

**Fig 13:**
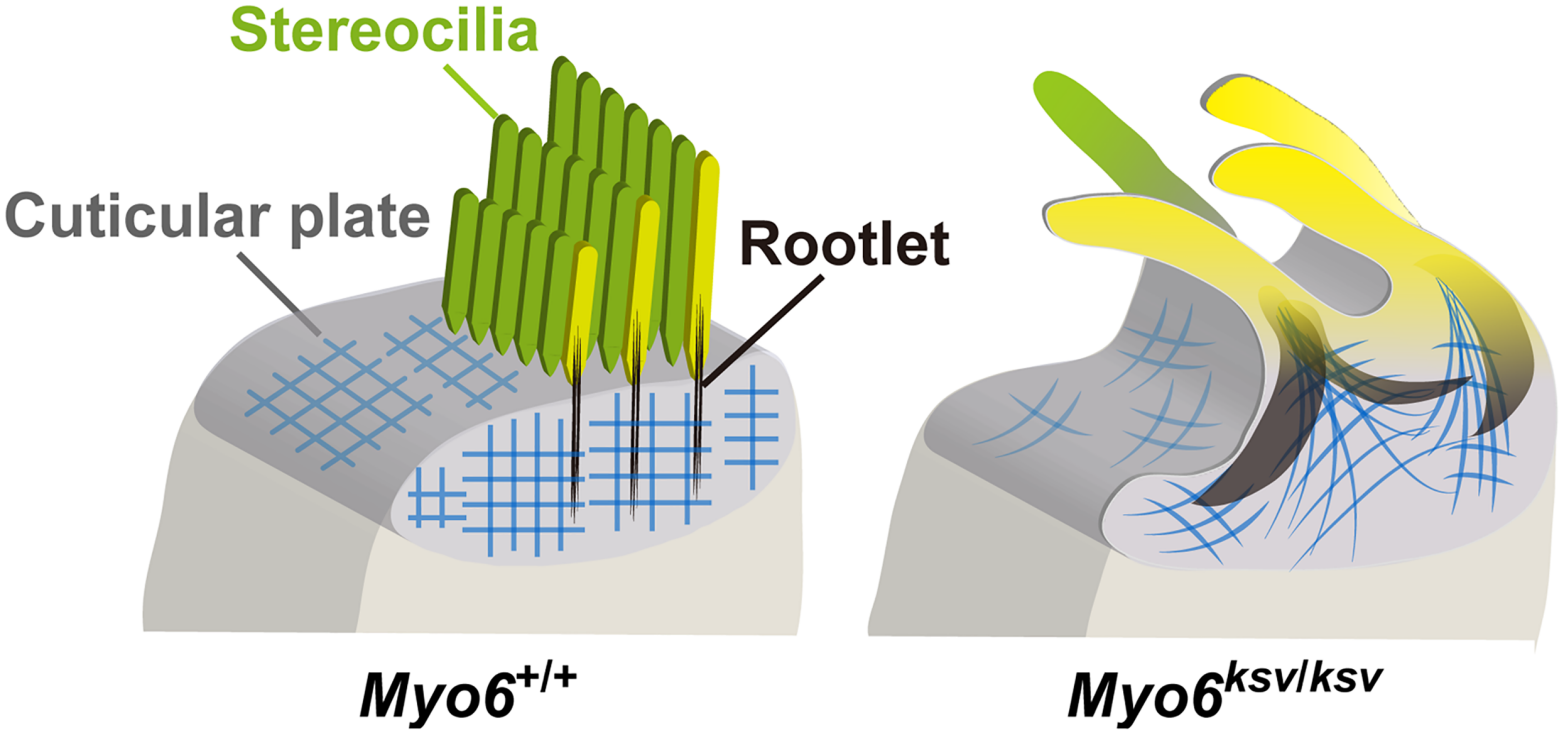
Schematic representation of the stereocilia in *Myo6* mutant mice shows how stereocilia fuse. Illustrations show apical surfaces of IHCs in +/+ (left) and *Myo6* mutant (*ksv*/*ksv*) (right) mice as the model for the normal and abnormal architectures of the stereociliary base. In +/+ mice, stereocilia have dense rootlets (black) that extend through the taper region to anchor them into the actin mesh (blue) of the cuticular plate. The structures are maintained when MYO6 is normally expressed in the stereociliary taper, cuticular plate and cytoplasm (left), but the appreciable reduction of MYO6 by *ksv* mutations leads to stereociliary fusion accompanied by the deformation of the cuticular plates and the extension of the rootlets (right).

Here, we showed the phenotypes occurring during the window of stereociliary fusion in the cochlear hair cells of *ksv*/*ksv* mice, by using electron microscopy analyses and immunohistochemistry. In both the IHCs and OHCs, clear abnormalities in the rotation of the stereociliary bundles and the mislocalization of the kinocilia were first observed in the *ksv*/*ksv* mice at P0 (Fig 7B and S2 Fig). The orientation defects of the stereocilia and kinocilia have previously been reported in several *Myo6* homozygous mutants [20, 23, 35, 48] and heterozygotes of an N-ethyl N-nitrosourea (ENU)-induced *Myo6*^*Tlc*^ mutant allele [19], thus suggesting that *Myo6* mutants commonly have planar cell polarity (PCP) defects. Although PCP defects were detected in many hair cells, similar differentiation and ranking of the stereociliary bundles at the lateral edge with +/+ mice were observed in *ksv*/*ksv* mice at P0–P2 (Figs 7A, 7B, 9A–9D and 10A–10F and S3 Fig). In the hair cells of the *ksv*/*ksv* mice, the bases of the stereocilia that converged at the lateral edge showed bulging (Figs 9A, 9B, 9D, 10A, 10B and 10D and S3 Fig). Similar phenotypes have been observed in the hair cells of several *Myo6* mutants [19, 23, 35] at early postnatal stages (P0–P3), thus suggesting that the rising of the cuticular plate membranes is a common event at the beginning of stereociliary fusion. Moreover, we confirmed by immunohistochemistry using cuticular plate-specific markers that the bulging of the stereociliary base was accompanied by fusion with the raised cuticular plate membrane (Fig 11). The cuticular plate is a dense matrix of F-actin, but it is a distinct structure with a unique organization of F-actin from the stereocilia [5]. Our results suggested that the drastic decrease in MYO6 disrupts the F-actin structures between the stereocilia and the cuticular plates of the hair cells (Fig 13).

Similar defects were detected in vestibular hair cells of *ksv*/*ksv* mice. We observed the PCP defects (Fig 8C) and bulged stereocilary bases (Figs 1D’ and 8D’) and thus predicted that these phenotypes reflect events for stereociliary fusion caused by decreased MYO6. However, the stereociliary fusion in vestibular hair cells of *ksv*/*ksv* mice at early postnatal stages was difficult to explain, as compared with those of cochlear hair cells. In particular, the stereociliary fusion and bulging of the stereociliary base appeared to delay development, in vestibular hair cells compared with cochlear hair cells (Figs 1D, 1D’, 7E, 7F, 8D and 8D’). A previous study has demonstrated that severe stereociliary fusion in the vestibular hair cells of *Myo6* mutant mice develops at 8 weeks of age [35]. Although we restricted the phenotypic analysis to the early postnatal stage, phenotypic analysis at older stages may be required to understand the events in the process associated with stereociliary fusion in vestibular hair cells of *Myo6* mutants, including *ksv*/*ksv* mice.

The phenotypes of the cuticular plate membranes between the IHCs and OHCs were also slightly different in *ksv*/*ksv* mice. The raised cuticular plate membranes in the IHCs occurred earlier than those in the OHCs and were more severe (Figs 9A, 9B, 10A, 10B, 11B, 11D, 11F and 11H). In mice, the hair bundle of the IHCs is well developed in comparison with that of the OHCs at early postnatal stages, thus suggesting that the growth of the IHCs is faster [49]. Therefore, the phenotypic differences may be related to the different maturities of the two types of cochlear hair cells at the start of the raised cuticular plate membranes and stereociliary fusion. Moreover, we found that the cuticular plates caved in and then incorporated with the stereocilia bundles in the cochlear hair cells of *ksv*/*ksv* mice at early postnatal stages (Fig 10D–10F and S4 Fig). In addition, we detected a connection via links between the incorporated stereociliary bundle and the cuticular plate membranes (Fig 10F’ and 10G). The links may be stereocilial (tip links, horizontal top connectors, shaft connectors and ankle links) and/or kinocilial (S5 Fig) [3, 50, 51].

Stereocilia incorporation was more often observed in OHCs versus IHCs of *ksv*/*ksv* mice with IHC observations of stereocilia incorporation being rare. In the OHCs, a thick sheath is formed that surrounds the stereociliary base (or rootlets) in the cuticular plate [4]. We predict that the stereocilia bundles are incorporated into the sheath. The cuticular plate of the OHCs is thicker than that of the IHCs [5, 52]. Interestingly, the thickening of the cuticular plates occurs during the early postnatal stages (P0–P5) [53]. This morphological change in the cuticular plate in the OHCs may be associated with the incorporation within the cuticular plates of the stereocilia in *ksv*/*ksv* mice.

In addition, we found that the *ksv*/*ksv* mice showed an abnormal elongation of the rootlets (Fig 12), which are also distinct F-actin structures of the stereociliary base [4, 5]. The rootlet phenotype of the IHCs appeared to be more severe than that of the OHCs of *ksv*/*ksv* mice. However, we predict that the degree of damage to the rootlets of the IHCs and OHCs is similar in the *ksv*/*ksv* mice because the rootlet length of the IHCs is approximately twice that of the OHCs [4]. The rootlets are embedded in and associated with an F-actin meshwork in the cuticular plate [54, 55]; therefore, the bulging and rising of the cuticular plates in *Myo6* mutants may occur, in which the F-actin meshwork of the cuticular plates are pulled toward the stereocilia bundles, and this is followed by the elongation of the rootlets (Fig 13), thus suggesting that the elongation of the rootlets may be major event in the fusion of the stereocilia in *ksv*/*ksv* mice. An actin-binding protein, TRIOBP, is the only protein that has been confirmed to play an essential role in the formation of rootlets, because *Triobp* KO mice cannot form rootlets [28]. Moreover, *Triobp* KO mice show progressive stereociliary fusion in both IHCs and OHCs [28]. The phenotypes of the rootlets between the *Myo6* and *Triobp* mutant mice are completely different, but they support the concept that the degeneration of the rootlets leads to the stereociliary fusion in the hair cells of mice.

This phenotype of *ksv*/*ksv* mice suggests that MYO6 regulates and maintains the rootlet length. Several proteins have been found to localize to rootlets [4, 5, 32], but the molecules responsible for their development and maintenance are not well known. There is probably a protein complex including TRIOBP, tapering proteins and a rootlet-specific crosslinking protein that has not yet been identified. Although the precise molecular mechanism by which MYO6 regulates the maintenance of rootlets through the interaction with the protein complex of the stereociliary base remains to be confirmed, we speculate that MYO6 directly or indirectly interacts with the complex and may play a role as a cargo transporter that transports the member(s) of the complex to the rootlets.

## Supporting information

S1 Fig Cellular localization of *ksv* mutant isoforms of MYO6 in COS7 cells. A–F. Single transfections of GFP-MYO6 (wild-type, A), GFP-MYO6^G360_F460del^ (B), GFP-MYO6^E461K^ (C), DsRed-MYO6 (D), DsRed-MYO6^G360_F460del^ (E), and DsRed-MYO6^E461K^ (F) constructs in COS7 cells. The fluorescence images show the expressed GFP-tagged MYO6 constructs (green), DsRed-tagged MYO6 constructs (red) and DAPI staining (blue). G–J. Co-transfection of GFP-MYO6 with DsRed-MYO6^G360_F460del^ (G), GFP-MYO6 with DsRed-MYO6^E461K^ (H), DsRed-MYO6 with FLAG-tagged MYO6^E461K^ (green) (I), and FLAG-tagged MYO6 with DsRed-MYO6^G360_F460del^ (J) constructs in COS7 cells. Scale bars = 5 μm.
(TIF)

S2 Fig Quantification of kinocilia positions in the OHCs and IHCs of +/+ and *ksv*/*ksv* mice. A and B. Surface images of the hair cells from the middle area of the cochlea in +/+ (A) and *ksv*/*ksv* (B) mice at P0 visualized by phalloidin (gray) and β-tubulin (green) staining. C. Schematic diagrams of kinocilia positions in OHCs and IHCs of +/+ and *ksv*/*ksv* mice. The kinocilia positions (dark gray small circles) are mapped onto a cuticular plate (right gray circle), on the basis of surface images (A and B). D. Distributions of the kinocilia positions in OHCs and IHCs of +/+ and *ksv*/*ksv* mice at P0. Color shadings of the *x*-axis indicate kinocilia positions, with black corresponding to normal positions (0°–15°) and light gray to abnormal positions.
(TIF)

S3 Fig Stereociliary phenotypes of *ksv*/*ksv* mice at P2. SEM images showing stereocilia from the middle (top) and base (bottom) areas of the cochlea. Scale bars = 5 μm.
(TIF)

S4 Fig Incorporation of stereociliary bundles in the cochlear hair cells in *ksv*/*ksv* mice during the early postnatal stages. A and B. Stereociliary phenotypes of *ksv*/*ksv* mice at P4 (A) and P7 (B). SEM images showing stereocilia in the hair cells from the base areas of the cochlea. Highly magnified images of surfaces of the hair cells (dotted boxes) in A and B are shown in each right panel (A’ and B’). Open arrows indicate the pocket-like structures of cuticular plates identified in the bases of the incorporated stereociliary bundles. Scale bars = 1 μm (A and B) and 300 nm (A’ and B’). C. Appearance ratio of the hair cells, which are the incorporated stereociliary bundles in the cuticular plate in *ksv*/*ksv* mice. Bars show average ratios with SD (error bars) of the IHCs and OHCs, which are the stereociliary bundles incorporated into the cuticular plate observed in SEM images of the hair cells from the apex, middle and base area of the cochlea in *ksv/ksv* mice at P2, P4 and P7.
(TIF)

S5 Fig SEM images of stereocilia, kinocilia and links as the connectors in hair bundles. A and B. Ventral (A) and dorsal (B) views of normal stereocilia of OHCs at early postnatal stages. Highly magnified images in dotted boxes of A and B show the stereocilial (A’) and kinocilial (B’) links, respectively. Scale bars = 1 μm (A and B) and 300 nm (A’ and B’). C. Diagram illustrating the stereocilial links (tip link, top connector, shaft connector, ankle link) and kinocilial link organized in the stereocilia bundles of the organ of Corti.
(TIF)

S1 Table Oligonucleotides for PCR Used for RT-PCR and the generation of DNA constructs in this study. (XLSX)

S2 Table Antibodies used in this study. (XLSX)
