# Supplementary figures and images for: A novel splice site mutation of myosin VI in mice leads to stereociliary fusion caused by disruption of actin networks in the apical region of inner ear hair cells
Source: PLoS One. 2017 Aug 23;12(8):e0183477. doi: 10.1371/journal.pone.0183477 (PMC5568226; doi:10.1371/journal.pone.0183477)

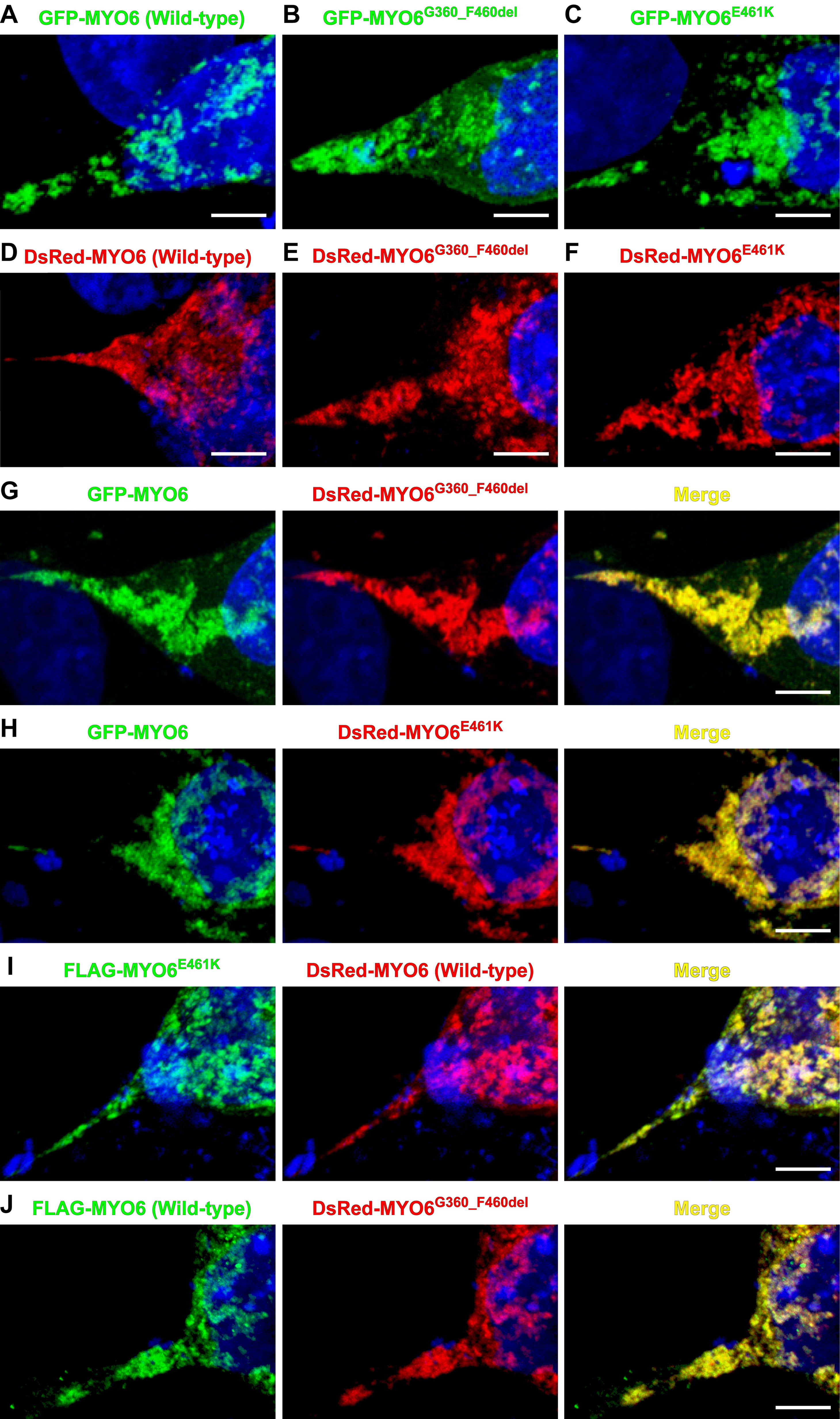

Supplement: S1 Fig — A–F. Single transfections of GFP-MYO6 (wild-type, A), GFP-MYO6G360_F460del (B), GFP-MYO6E461K (C), DsRed-MYO6 (D), DsRed-MYO6G360_F460del (E), and DsRed-MYO6E461K (F) constructs in COS7 cells. The fluorescence images show the expressed GFP-tagged MYO6 constructs (green), DsRed-tagged MYO6 constructs (red) and DAPI staining (blue). G–J. Co-transfection of GFP-MYO6 with DsRed-MYO6G360_F460del (G), GFP-MYO6 with DsRed-MYO6E461K (H), DsRed-MYO6 with FLAG-tagged MYO6E461K (green) (I), and FLAG-tagged MYO6 with DsRed-MYO6G360_F460del (J) constructs in COS7 cells. Scale bars = 5 μm. (TIF) [file pone.0183477.s001.tif]

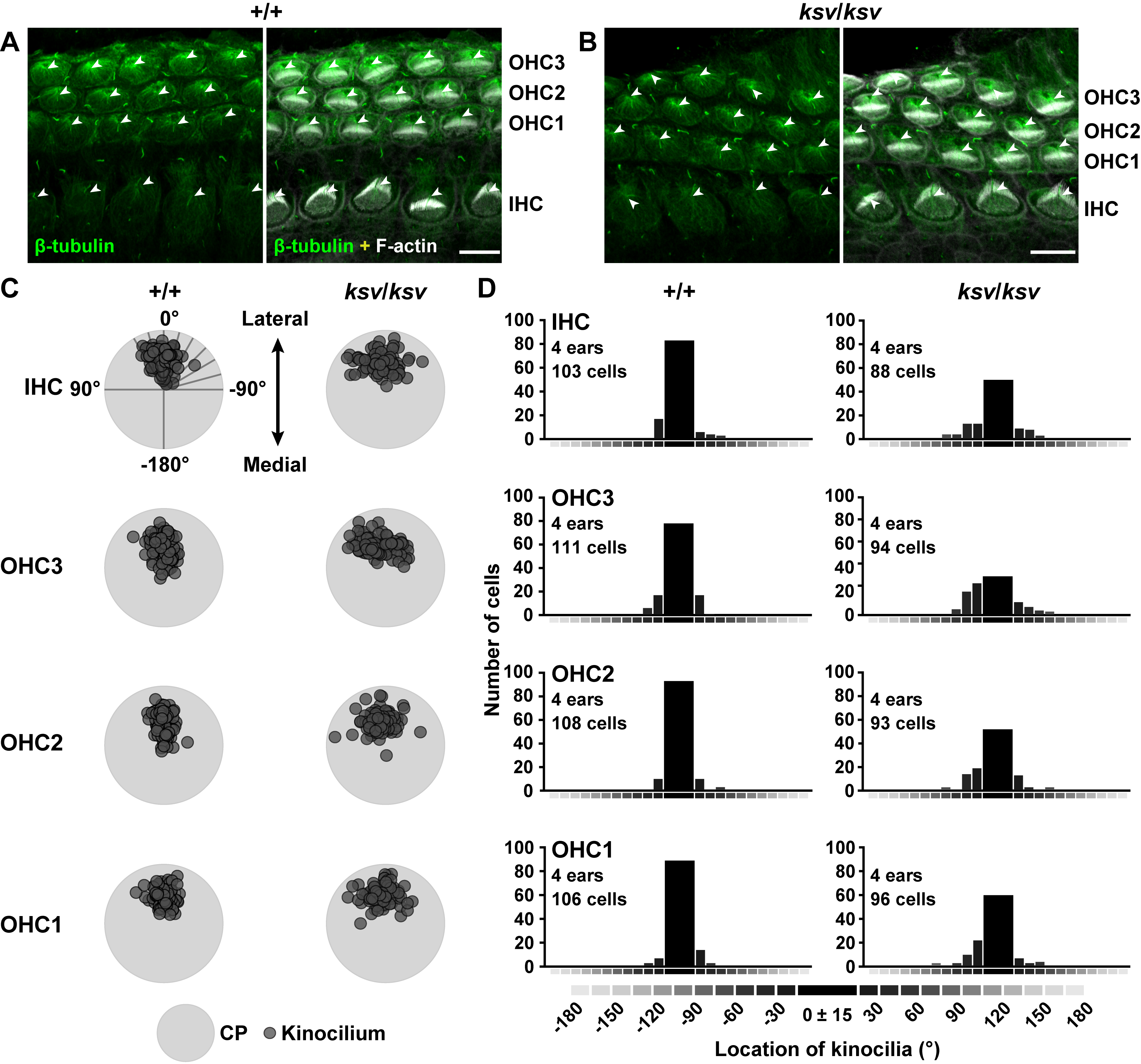

Supplement: S2 Fig — A and B. Surface images of the hair cells from the middle area of the cochlea in +/+ (A) and ksv/ksv (B) mice at P0 visualized by phalloidin (gray) and β-tubulin (green) staining. C. Schematic diagrams of kinocilia positions in OHCs and IHCs of +/+ and ksv/ksv mice. The kinocilia positions (dark gray small circles) are mapped onto a cuticular plate (right gray circle), on the basis of surface images (A and B). D. Distributions of the kinocilia positions in OHCs and IHCs of +/+ and ksv/ksv mice at P0. Color shadings of the x-axis indicate kinocilia positions, with black corresponding to normal positions (0°–15°) and light gray to abnormal positions. (TIF) [file pone.0183477.s002.tif]

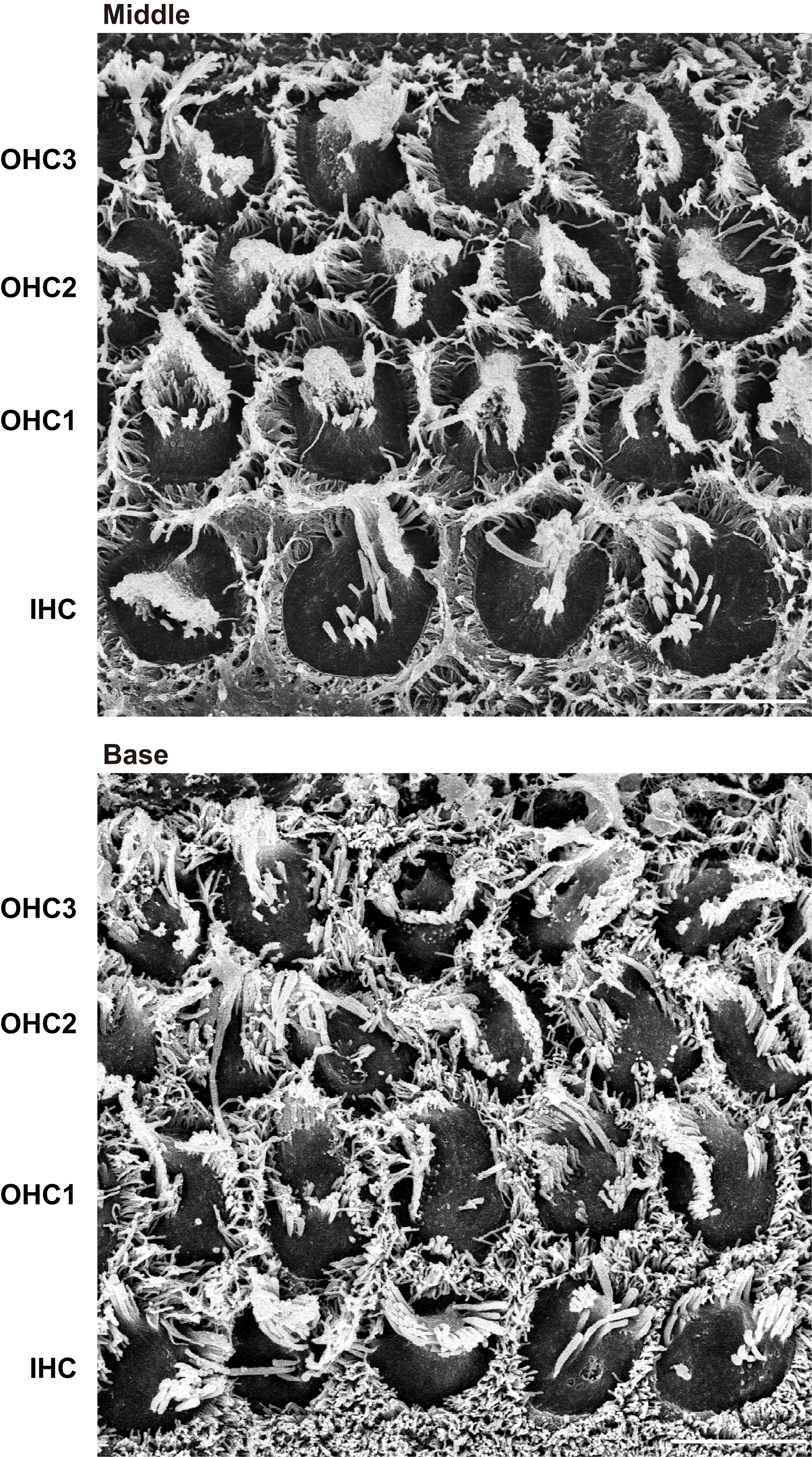

Supplement: S3 Fig — SEM images showing stereocilia from the middle (top) and base (bottom) areas of the cochlea. Scale bars = 5 μm. (TIF) [file pone.0183477.s003.tif]

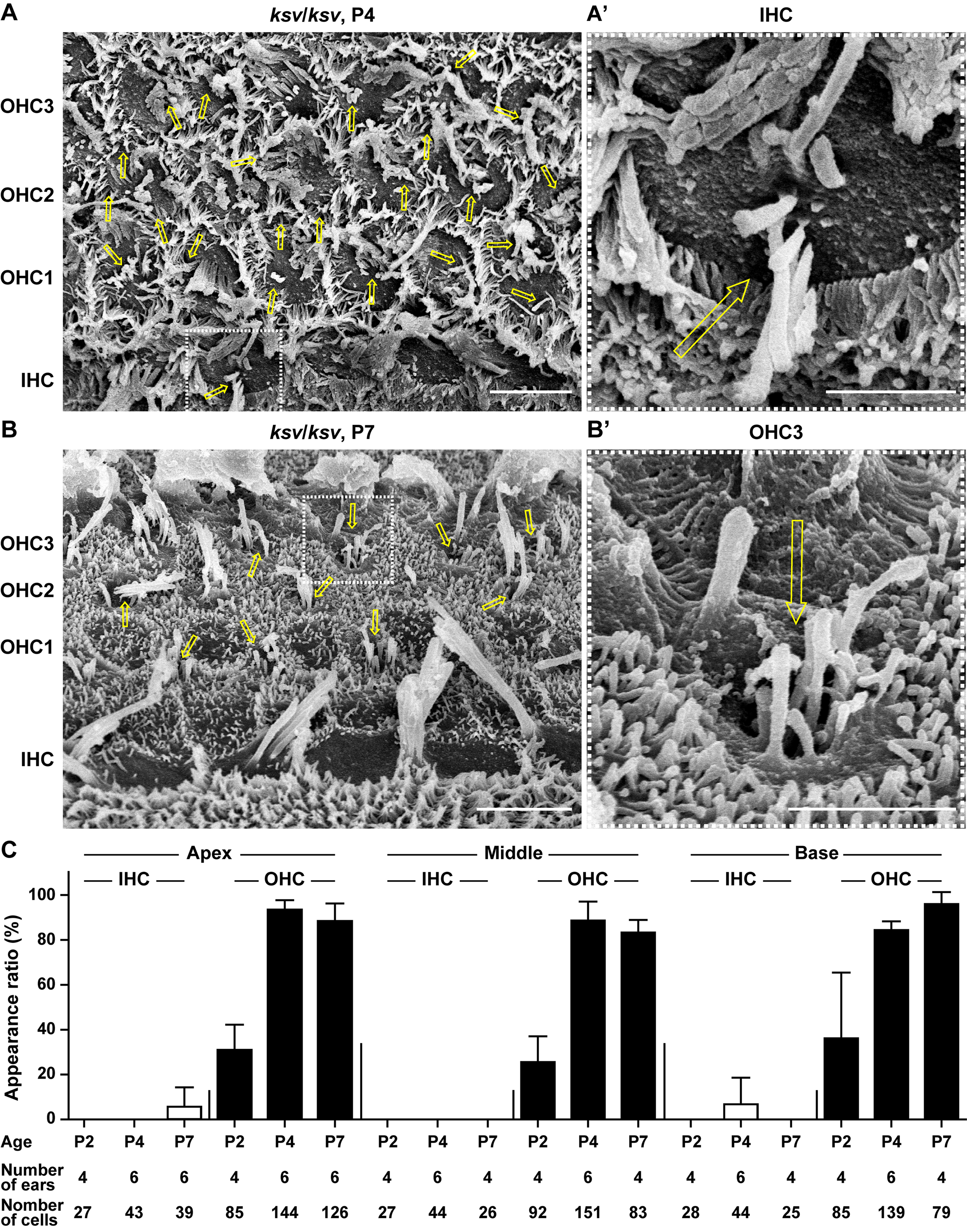

Supplement: S4 Fig — A and B. Stereociliary phenotypes of ksv/ksv mice at P4 (A) and P7 (B). SEM images showing stereocilia in the hair cells from the base areas of the cochlea. Highly magnified images of surfaces of the hair cells (dotted boxes) in A and B are shown in each right panel (A’ and B’). Open arrows indicate the pocket-like structures of cuticular plates identified in the bases of the incorporated stereociliary bundles. Scale bars = 1 μm (A and B) and 300 nm (A’ and B’). C. Appearance ratio of the hair cells, which are the incorporated stereociliary bundles in the cuticular plate in ksv/ksv mice. Bars show average ratios with SD (error bars) of the IHCs and OHCs, which are the stereociliary bundles incorporated into the cuticular plate observed in SEM images of the hair cells from the apex, middle and base area of the cochlea in ksv/ksv mice at P2, P4 and P7. (TIF) [file pone.0183477.s004.tif]

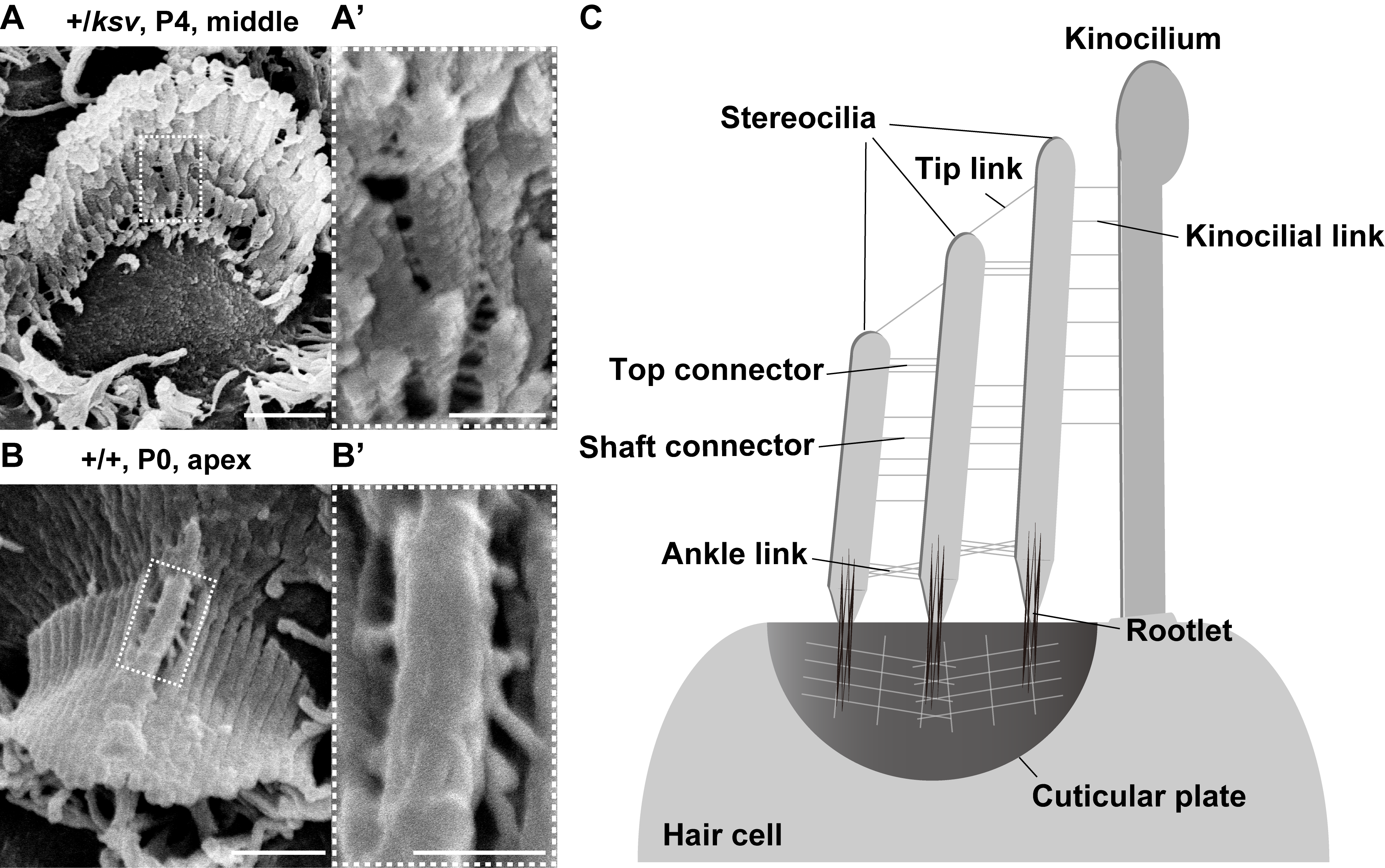

Supplement: S5 Fig — A and B. Ventral (A) and dorsal (B) views of normal stereocilia of OHCs at early postnatal stages. Highly magnified images in dotted boxes of A and B show the stereocilial (A’) and kinocilial (B’) links, respectively. Scale bars = 1 μm (A and B) and 300 nm (A’ and B’). C. Diagram illustrating the stereocilial links (tip link, top connector, shaft connector, ankle link) and kinocilial link organized in the stereocilia bundles of the organ of Corti. (TIF) [file pone.0183477.s005.tif]
